# MYRF Is a Membrane-Associated Transcription Factor That Autoproteolytically Cleaves to Directly Activate Myelin Genes

**DOI:** 10.1371/journal.pbio.1001625

**Published:** 2013-08-13

**Authors:** Helena Bujalka, Matthias Koenning, Stacey Jackson, Victoria M. Perreau, Bernard Pope, Curtis M. Hay, Stanlislaw Mitew, Andrew F. Hill, Q. Richard Lu, Michael Wegner, Rajini Srinivasan, John Svaren, Melanie Willingham, Ben A. Barres, Ben Emery

**Affiliations:** 1 Department of Anatomy and Neuroscience and the Centre for Neuroscience Research, University of Melbourne, Parkville, Australia; 2 Department of Computing and Information Systems and Victorian Life Sciences Computation Initiative (VLSCI), University of Melbourne, Parkville, Australia; 3 Department of Biochemistry and Molecular Biology, Bio21 Molecular Science and Biotechnology Institute, University of Melbourne, Parkville, Australia; 4 Department of Developmental Biology and Kent Waldrep Foundation Center for Basic Neuroscience Research on Nerve Growth and Regeneration, University of Texas at Austin, Austin, Texas, United States of America; 5 Institute for Biochemistry, University Erlangen-Nuernberg, Germany; 6 Waisman Center, University of Wisconsin–Madison, Madison, Wisconsin, United States of America; 7 Department of Neurobiology, Stanford University, Stanford, California, United States of America; 8 Florey Institute of Neuroscience and Mental Health, Parkville, Australia; University of Edinburgh, United Kingdom

## Abstract

The myelination of axons is a crucial step during vertebrate central nervous system (CNS) development, allowing for rapid and energy efficient saltatory conduction of nerve impulses. Accordingly, the differentiation of oligodendrocytes, the myelinating cells of the CNS, and their expression of myelin genes are under tight transcriptional control. We previously identified a putative transcription factor, Myelin Regulatory Factor (*Myrf*), as being vital for CNS myelination. *Myrf* is required for the generation of CNS myelination during development and also for its maintenance in the adult. It has been controversial, however, whether *Myrf* directly regulates transcription, with reports of a transmembrane domain and lack of nuclear localization. Here we show that *Myrf* is a membrane-associated transcription factor that undergoes an activating proteolytic cleavage to separate its transmembrane domain-containing C-terminal region from a nuclear-targeted N-terminal region. Unexpectedly, this cleavage event occurs via a protein domain related to the autoproteolytic intramolecular chaperone domain of the bacteriophage tail spike proteins, the first time this domain has been found to play a role in eukaryotic proteins. Using ChIP-Seq we show that the N-terminal cleavage product directly binds the enhancer regions of oligodendrocyte-specific and myelin genes. This binding occurs via a defined DNA-binding consensus sequence and strongly promotes the expression of target genes. These findings identify *Myrf* as a novel example of a membrane-associated transcription factor and provide a direct molecular mechanism for its regulation of oligodendrocyte differentiation and CNS myelination.

## Abstract 2

Oligodendrocyte development and myelination rely on an unusual membrane-associated transcription factor that shares functional domains with bacteriophage proteins.

## Author Summary

Oligodendrocytes are a highly specialized cell type that surround axons of the vertebrate central nervous system with myelin, electrically insulating them and allowing rapid and energy-efficient propagation of nerve signals. We previously identified a protein, MYRF, that is required for the final stages of oligodendrocyte differentiation and myelination. Although we proposed that MYRF might act as a transcription factor, it remains uncertain whether this is true, given that MYRF and related proteins contain a transmembrane domain that might preclude localization to the nucleus. Here, we show that the MYRF protein undergoes an activating cleavage event to release the functional transcription factor from the transmembrane domain that otherwise anchors it to the endoplasmic reticulum. Unexpectedly, this cleavage event is mediated by a portion of MYRF that is related to a self-cleaving domain found in bacteriophage proteins. This distinguishes it from other membrane-associated transcription factors that are cleaved via regulated proteolysis within the membrane bilayer. We find that the N-terminal product of MYRF cleavage directly binds to a wide range of genes involved in myelination, stimulating their expression. Many of these MYRF binding sites identify previously uncharacterized enhancers for these myelin genes.

## Introduction

Oligodendrocytes are the myelinating cells of the vertebrate CNS; their development and the ensheathment of receptive neuronal axons are vital for the rapid propagation of nerve impulses. Accordingly, the differentiation of oligodendrocyte progenitor cells (OPCs) into oligodendrocytes and their subsequent myelination of axons are highly regulated processes. At the transcriptional level, the factors involved in the development of the oligodendrocyte lineage have been relatively well characterized. The transcription factor *Olig2* is required for specification of OPCs from subventricular zone precursor cells, at least within ventral regions of the CNS [1],[2]. *Olig2* is continually expressed in the lineage and has later roles in directing the chromatin-remodeling enzyme *Brg1* to regulatory elements of target genes during differentiation [3]. A number of other transcription factors are subsequently required for the successful differentiation of OPCs into myelinating oligodendrocytes including *Olig1* [4], *Nkx2.2* [5], *Ascl1/Mash1* [6], *Zfp191* [7], and *Sox10* [8],[9].

We recently identified Myelin Regulatory Factor (*Myrf*; previously known as *Gene Model 98* and *MRF*) as a transcript that is highly induced during oligodendrocyte differentiation and absent in other CNS cell types [10]. Based on the MYRF protein containing a putative DNA-binding domain (DBD) with homology to that of the yeast transcription factor *Ndt80* [11],[12], we proposed that *Myrf* might act as a direct transcriptional regulator of CNS myelination. Consistent with this hypothesis, conditional ablation of *Myrf* causes severe CNS dysmyelination, with oligodendrocytes stalling at the pre-myelinating stage and showing severe deficits in myelin gene expression [13]. Inducible ablation of *Myrf* in mature oligodendrocytes of the adult CNS also causes a rapid down-regulation of myelin gene expression followed by a gradual degeneration of CNS myelin [14]. Unlike previously described transcription factors *Olig1*, *Olig2*, *Sox10*, *Nkx2.2*, and *Ascl1*, *Myrf* is expressed only at the postmitotic stage of the oligodendrocyte lineage, suggesting that its induction is a key step in the regulation of myelination.

While these results identified a vital role for *Myrf* in the generation and maintenance of CNS myelin, they did not address the molecular mechanisms by which it acts. Notably, the assignment of *Myrf* as a transcription factor was recently questioned based on a lack of nuclear localization of the *C. elegans* ortholog, *pqn-47*, with *pqn-47* and *Myrf* instead proposed to have a role in secretion of proteins from the endoplasmic reticulum/Golgi [15]. Consistent with this, the MYRF protein contains at least one hydrophobic region that originally led to the human ortholog *MYRF/C11Orf9* being classed as a probable transmembrane protein [16]. Together, these findings raise the question of whether *Myrf* and its orthologs promote myelination through the direct regulation of key myelin genes, or whether they may act via other mechanisms involving the membrane and myelin protein trafficking system previously implicated in myelination [17].

Here, we investigate the molecular mechanisms by which *Myrf* mediates oligodendrocyte differentiation and myelination. We find that the MYRF protein is subject to autoproteolytic cleavage within a domain related to bacteriophage tail spike proteins. This cleavage yields an N-terminal nuclear-targeted fragment containing the DBD, and is required for MYRF's promotion of myelin gene expression. Through ChIP-Seq analysis and luciferase assays we show that MYRF binds the *cis*-regulatory elements of multiple oligodendrocyte-specific genes involved in myelination. This binding occurs via a defined DNA consensus sequence and strongly promotes transcription from these elements. These findings establish *Myrf* as a membrane-associated transcription factor with a direct role in stimulating myelin gene expression.

## Results

### *In Silico* Prediction of MYRF Features

**Figure 1:**
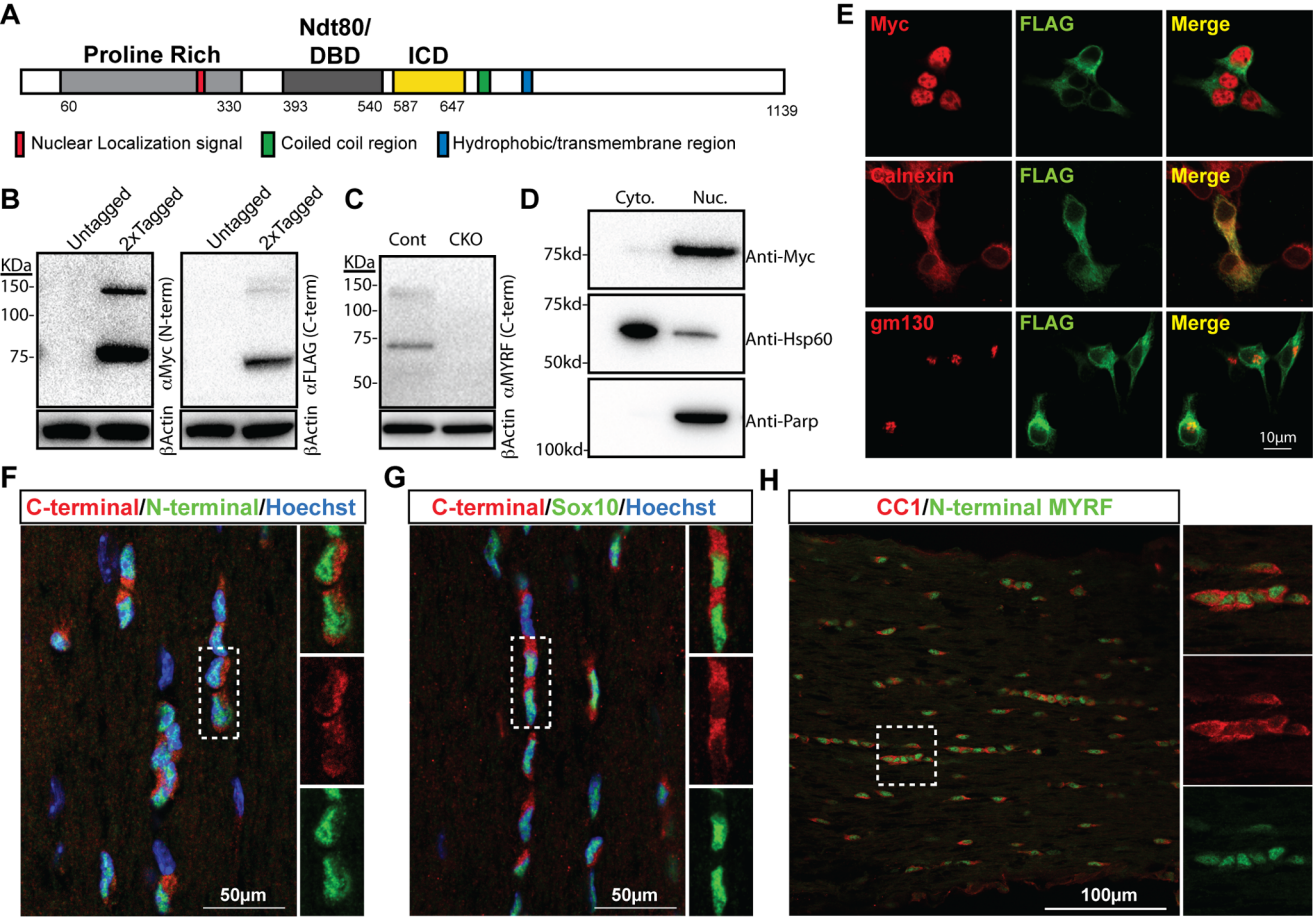
The MYRF protein is subject to posttranslational cleavage. (A) Schematic of the MYRF protein showing positions of the predicted NLS, Ndt80-like DBD, ICD, coiled coil region, and transmembrane region. (B) Western blot analysis of a double tagged (N-terminal Myc, C-terminal FLAG tagged) or untagged MYRF expression construct in 293T cells. Probing with anti-Myc or anti-FLAG reveals the presence of a ∼140 kDa full-length fragment and truncated cleavage products. (C) Western blot of control and *Myrf* CKO cultured mouse oligodendrocyte lysates with anti-C-terminal MYRF mAb. (D) Cell fractionation experiment showing that the majority of Myc-tagged N-terminal cleaved MYRF product is present in the nucleus of 293T cells (Anti-Hsp60 and anti-Parp used for cytoplasmic and nuclear controls, respectively). (E) CG-4 cells transfected with the Myc-MYRF-FLAG construct were co-stained for anti-FLAG and either anti-Myc, anti-Calnexin, or anti-golgi matrix protein 130 (gm130). (F–H) Adult mouse optic nerve stained with the anti-N-terminal-MYRF and anti-C-terminal-MYRF-mab antibodies (F), anti-C-terminal-MYRF-mab and anti-Sox10 (G), or anti-N-terminal-MYRF-mab and CC1 (H).

In spite of its clear role in regulating CNS myelination, little is known about *Myrf* at the protein level. To learn more about the features and likely function of the MYRF protein, we identified functional domains in its 1,139 aa sequence [13] by homology analysis (see Materials and Methods). Predicted features (illustrated in Figure 1A) include the previously described proline-rich region (residues 60–330) and putative Ndt80-like DBD (residues 393–540). In agreement with Stohr et al. [16], a transmembrane region was predicted by a number of programs at residues 767–789. In addition, several targeting motif predictors (ELM and NucPred) identified a likely nuclear localization signal (NLS) at residues 252–258. A putative coiled coil domain was identified at residues ∼685–706, though with only moderate confidence. HHpred searches as well as the NCBI conserved domain search function identified a region of similarity to the intramolecular chaperone domain (ICD) of the bacteriophage tail spike proteins at residues 587–647 (see below for further details). These predicted features and the confidence scores associated with them are listed in Table S1.

### MYRF Protein Is Cleaved to Yield a Nuclear N-Terminal Fragment

The presence of both predicted transmembrane and DBDs raised the possibility that MYRF may be subject to proteolytic cleavage in a similar manner to membrane-associated transcription factors such as Notch and the SREBPs [18],[19]. We therefore performed Western blot analysis on a double-tagged MYRF expression construct (Myc-MYRF-FLAG) in 293T cells. Probing with both anti-Myc and anti-FLAG detected a faint ∼140 kda band corresponding to a full-length protein as well as more intense bands at ∼75 kda and 70 kda, respectively, corresponding to cleavage products (Figure 1B). Analysis of a number of truncated MYRF constructs indicated that this cleavage occurred several kDa past the C-terminal end of the predicted DBD (see Figure S1). To confirm that the cleavage was not an artifact of overexpression of the tagged protein or the cell line, we assessed the cleavage of the endogenous protein in primary mouse oligodendrocytes using a monoclonal antibody mapped to the C-terminal cleavage product of MYRF (anti-C-terminal-MYRF-mab). We found that the endogenous MYRF protein undergoes the same cleavage event, giving two bands of equivalent sizes to the anti-FLAG immunoblotting of the Myc-MYRF-FLAG construct. These bands were absent in lysates from MYRF conditional knockout (CKO; *Myrf^FL/FL^; Olig2^WT/Cre^*) oligodendrocytes (Figure 1C), confirming their identity.

Subcellular fractionation experiments indicated that the Myc-tagged N-terminal cleavage product was predominantly located in the nucleus (Figure 1D). To further investigate the subcellular localization of the cleavage products we transfected the CG-4 oligodendroglial cell line [20] with the Myc-MYRF-FLAG construct. Immunostaining confirmed that the Myc-tagged N-terminal product predominantly localized to the nucleus (though at higher exposure additional extranuclear staining was also apparent). In contrast, the FLAG-tagged C-terminus was excluded from the nucleus, predominantly co-localizing with the endoplasmic reticulum marker calnexin (Figure 1E). To determine localization *in vivo*, mouse optic nerve sections were stained with the anti-C-terminal-MYRF-mab and a rabbit polyclonal raised against the N-terminal region of MYRF (anti-N-terminal-MYRF). Co-staining with the two antibodies resulted in labeling of the same cells, with the anti-N-terminal antibody staining the nucleus and the anti-C-terminal monoclonal resulting in extranuclear staining of the same cells and in the majority of Sox10+ cells (Figure 1F and G). Double-staining with anti-N-terminal-MYRF and the mature oligodendrocyte marker CC1 confirmed that the MYRF-expressing cells were oligodendrocytes (Figure 1H). These results show that endogenous MYRF is subject to cleavage both *in vitro* and *in vivo*, resulting in nuclear translocation of the N-terminal domain only.

### Activating Cleavage of MYRF Occurs Via an Autoproteolytic ICD Related to Bacteriophage Tail Spike Proteins

**Figure 2:**
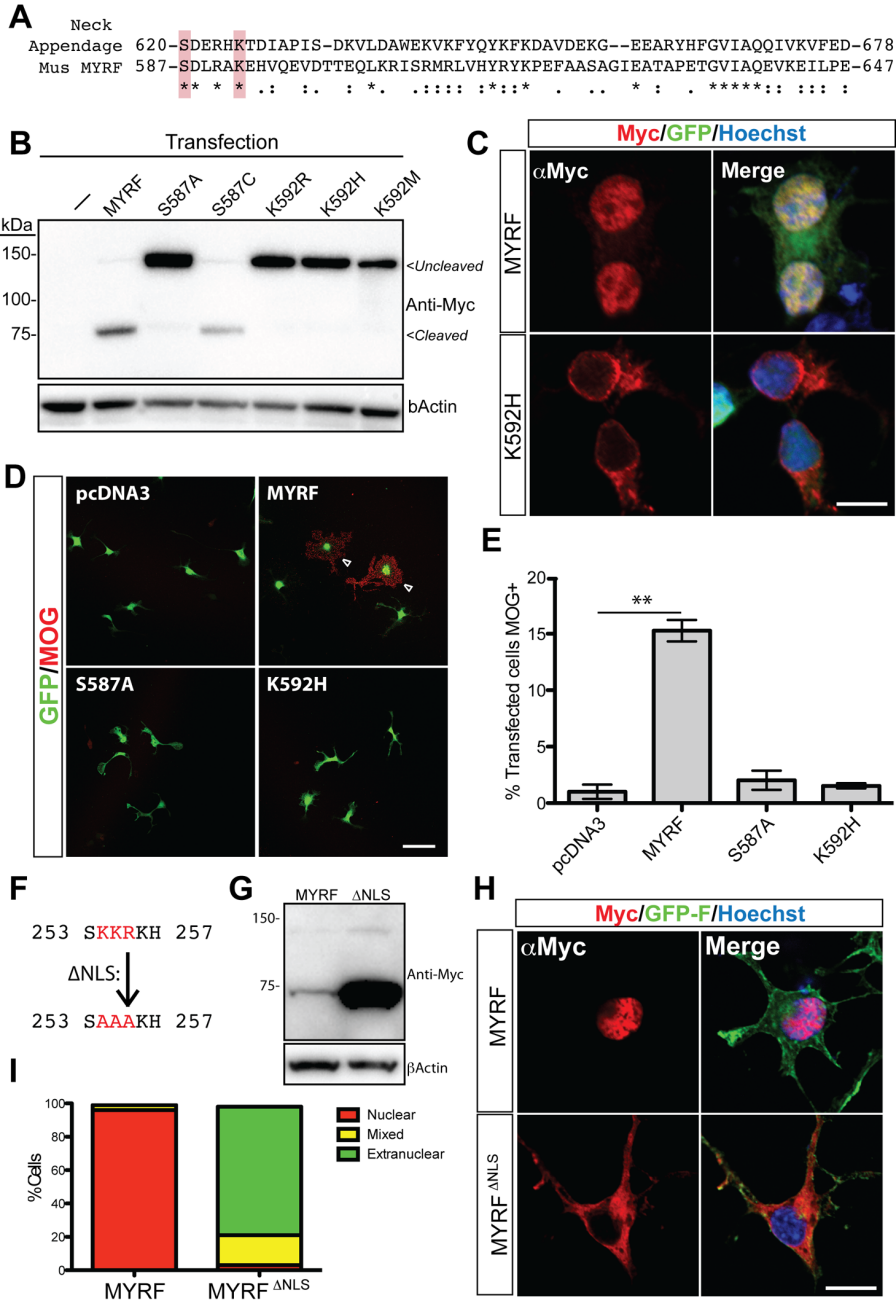
Autoproteolytic processing via the ICD and a NLS are required for nuclear localization of MYRF. (A) ClustalW2 alignment of the peptide sequence of MYRF and the ICD domain of the bacteriophage GA-1 Neck appendage protein. The serine/lysine dyad residues subjected to mutagenesis are highlighted. (B) Western blot analysis of un-mutated or ICD mutant (K592R, K592H, K592M) Myc-tagged MYRF constructs in 293T cells. (C) Immunofluorescence for the N-terminal Myc tagged MYRF and the K587H mutant in CG-4 cells. Prevention of the cleavage is associated with a loss of nuclear localization of the N-terminus. (D) Primary rat OPC cultures co-transfected with GFP and either empty vector (pcDNA3) or pcDNA3 containing MYRF or the S587A and K592H mutant constructs, stained for MOG. (E) Quantification of the percentage of transfected (GFP+) cells expressing MOG 48 h posttransfection in each condition. **p<0.01 by *t* test. (F) Predicted NLS within the proline-rich region of MYRF showing the KKR to AAA mutation in the Myc-MYRF^ΔNLS^ construct. (G) Western analysis of the Myc-MYRF and Myc-MYRF^ΔNLS^ construct; mutation of the NLS has no effects on the cleavage of MYRF, though it routinely led to an increase in protein levels. (H) Representative images of immunostaining for the N-terminal Myc tag showing shift from nuclear to extranuclear staining in the Myc-MYRF^ΔNLS^ construct. (I) Quantification of the proportion of predominantly nuclear, mixed, or predominantly extranuclear staining seen with each construct (100 cells assessed/condition). Scale bars, (C and H) 10 µm and (D) 50 µm.

We next sought to identify the mechanism of the cleavage event. HHpred and NCBI conserved domain searches using the region of MYRF that we predicted to contain the cleavage site as input (residues 546–763) revealed a region of significant homology to RCBS structural entries 3GUD and 3GW6 (with E-values of 2.8E-17 and 4.9E-12 for 3GUD3, respectively; see Table S1). These hits represent the ICD of the bacteriophage tail spike proteins, including the GP12 neck appendage and Endo-N-acetylneuraminidase (endosialidase) proteins. Alignment of MYRF and the bacteriophage neck appendage protein (Uniprot Q9FZW3) within this region of 61 amino acids revealed an amino acid identity of 21.3%, with 49.1% similarity (Figure 2A). The ICD mediates folding and subsequent autoproteolytic cleavage of these proteins to release a functional trimeric N-terminal fragment [21]–[23]. Although this protein domain has not to our knowledge been reported to mediate proteolytic cleavage of proteins in eukaryotes, the high degree of similarity between MYRF and the ICD of the bacteriophage tail spike proteins at a site closely matching the predicted cleavage site of MYRF was striking.

Autoproteolytic processing within the endosialidase ICD is dependent on a serine-lysine dyad that mediates cleavage at the serine residue [21],[22]. To assess whether this domain may also mediate cleavage of MYRF, we performed site-directed mutagenesis of the equivalent amino acids in MYRF (Figure 2A). Nonconservative mutation of S587 (S587A) or mutation of K592 (K592H, K592R, and K592M) in the Myc-MYRF-FLAG construct was sufficient to block the cleavage of MYRF as assessed by Western blot analysis; in contrast, the cleavage was preserved with a conservative mutation of the S587 residue (S587C) (Figure 2B). In addition to the absence of cleavage, the S587A, K592H, or K592R mutants were blocked from the nucleus (shown for the K592H mutation in Figure 2C), demonstrating that the cleavage is a prerequisite for nuclear localization of the protein. To determine the functional consequences of blocking cleavage we co-transfected primary rat OPCs in proliferative conditions with GFP and either empty vector (pcDNA3), or pcDNA containing Myc-MYRF-FLAG or the corresponding S587A and K592H mutant constructs. As previously described [13], forced expression of MYRF results in precocious expression of the mature marker Myelin Oligodendrocyte Glycoprotein (MOG) in a subset of cells within 48 h of transfection. In contrast, the S587A and K592H mutants did not increase MOG expression relative to the pcDNA3 control transfected cells (Figure 2D–E), confirming that the uncleavable mutants were unable to promote myelin gene expression.

The predicted NLS within the proline-rich region of MYRF (Figure 1A) was consistent with the observed nuclear localization of the N-terminal cleavage product. To assess whether the predicted NLS has a role in nuclear targeting of the N-terminal cleavage product, we mutated the putative NLS sequence in the Myc-MYRF construct (254KRR256 to 254AAA256; Myc-MYRF^ΔNLS^) (Figure 2F). Unlike mutation of the ICD, mutation of this putative NLS did not inhibit the cleavage of MYRF, however total levels of the Myc-MYRF^ΔNLS^ protein invariably appeared to be higher than the unmutated protein (Figure 2G). Immunostaining for the Myc-MYRF and Myc-MYRF^ΔNLS^ proteins showed that mutation of the NLS shifted the predominant localization of the N-terminal region from nuclear to extranuclear (Figure 2H and I), indicating that this NLS largely mediates the nuclear localization of the N-terminal portion of the protein.

These results demonstrate that the MYRF protein is cleaved via a domain related to the ICD chaperone domain of bacteriophage tail spike proteins to yield a nuclear-targeted N-terminal fragment consisting of the proline-rich region and DBD. The C-terminal cleavage product containing the transmembrane region is excluded from the nucleus.

### MYRF Directly Targets Genes Induced During Oligodendrocyte Differentiation

To examine whether the N-terminal cleavage product of MYRF binds directly to the genome in oligodendrocytes, we performed a ChIP-seq experiment immunoprecipitating the Myc-tagged MYRF construct and associated chromatin from cultured primary rat oligodendrocytes using anti-Myc. Previously published ChIP-Seq comparisons between expressed tagged and endogenous transcription factors have shown a high degree of agreement between the peaks identified with each method [24],[25]. Oligodendrocytes transfected with an untagged MYRF served as a negative control. ChIP-Seq analysis identified 2,102 peaks in the Myc-MYRF condition; 17 of these peaks had a corresponding peak in the untagged-MYRF control condition, indicating that they represented false positive peaks. These 17 peaks were removed from analysis leaving 2,085 peaks specific to the Myc-MYRF condition. No peaks were identified in the untagged-MYRF condition that did not have a corresponding peak in the Myc-MYRF condition. Genomic coordinates of all peaks are provided in Supporting Information S1.

We have previously shown that many of the oligodendrocyte-specific genes induced during differentiation (including abundant myelin genes such as *Mbp* and *Plp1*) are reliant on MYRF for their expression, however whether MYRF directly targets these genes is unknown. To determine whether MYRF binding sites are preferentially located proximal to these genes, we generated lists of the 200 most enriched genes in neurons, astrocytes, or postmitotic oligodendrocytes based on our published transcriptome database ([10], see Table S2 for the gene lists) and screened for the presence of MYRF peaks relative to their transcription start sites (TSSs).

**Figure 3:**
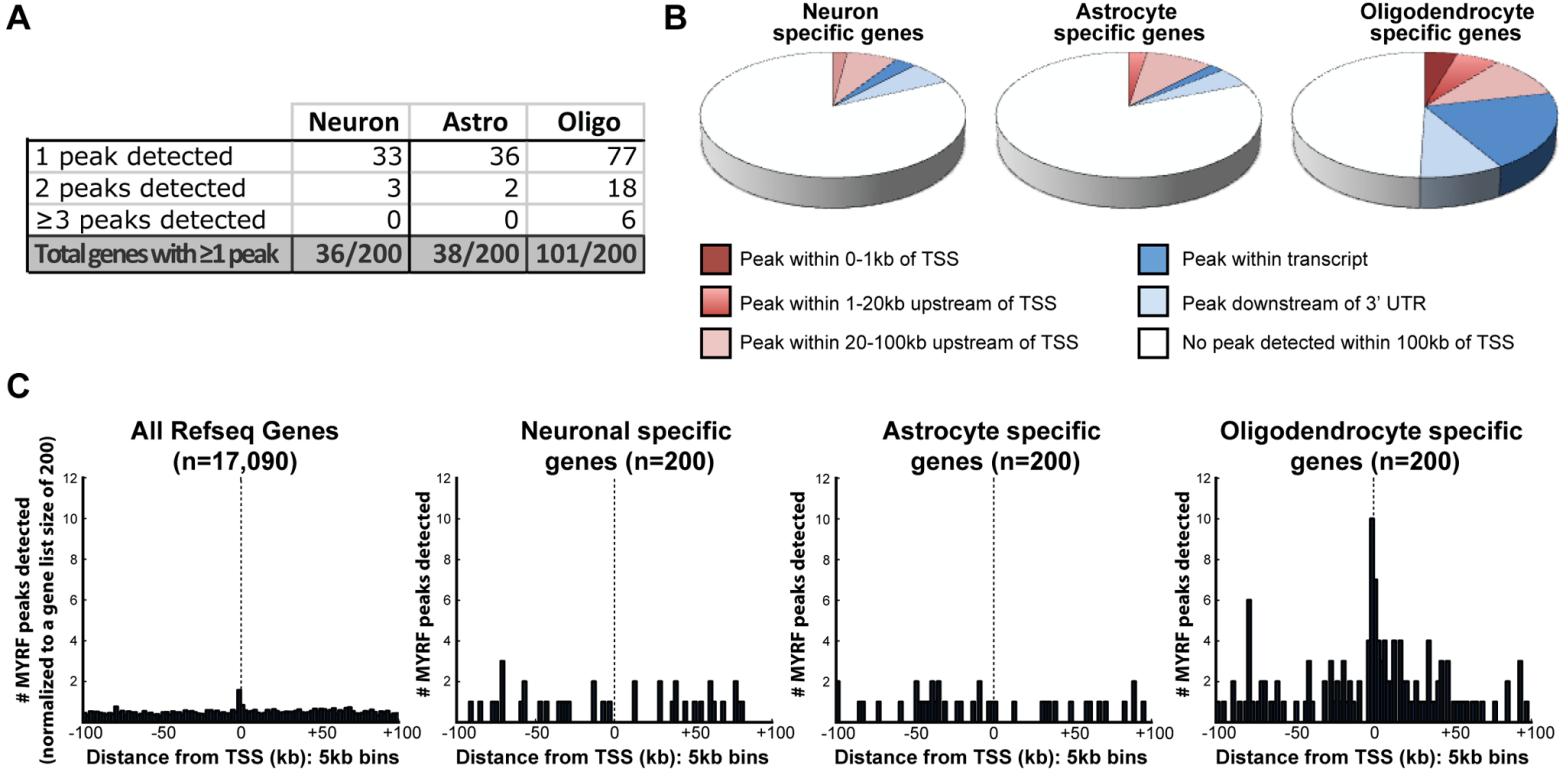
MYRF binds to regions of the genome surrounding oligodendrocyte enriched/myelin genes. (A) Number of the neuron-, astrocyte-, and oligodendrocyte-specific genes (from [10], 200 genes per list) with 1, 2, or ≥3 MYRF peaks detected within 100 kb of their TSS. (B) Pie charts showing the proportion of oligodendrocyte-, astrocyte-, or neuron-specific genes that have MYRF binding sites detected within 1 kb, 20 kb, or 100 kb upstream of the TSS, within the gene or downstream of the 3′ UTR (but still within 100 kb of the TSS). (C) Histograms showing the incidence of MYRF peaks relative to the TSS of either an unbiased list of rat Refseq genes (17,090 genes) or the neuron-, astrocyte-, or oligodendrocyte-specific genes. A modest increase in the incidence of MYRF binding is detectable proximal to the TSS of the unbiased gene list; a more pronounced increase in MYRF binding is observed around the TSS of oligodendrocyte-specific genes.

Based on the 2,085 observed MYRF peaks and an estimated size of the rat genome of 2.75 Gb [26], we would expect a peak to occur every 1.3 Mb on average. The number of peaks observed proximal to the TSSs of the 200 neuron and astrocyte-specific genes (which would not be expected to be regulated by MYRF) were relatively close to this expected background, with 39 and 40 peaks detected within 100 kb of their collective TSSs, respectively (expected = 30.8). In contrast, the oligodendrocyte-enriched genes showed a ∼3-fold increase in the density of peaks within 100 kb of their TSSs relative to the neuron and astrocyte genes, with multiple peaks detected for a number of genes (Figure 3A). This enrichment was particularly evident within 1 kb of the TSS and intronic regions of the oligodendrocyte-specific genes (Figure 3B) and was highly statistically significant (*p*<0.0001 by Chi-squared test).

**Figure 4:**
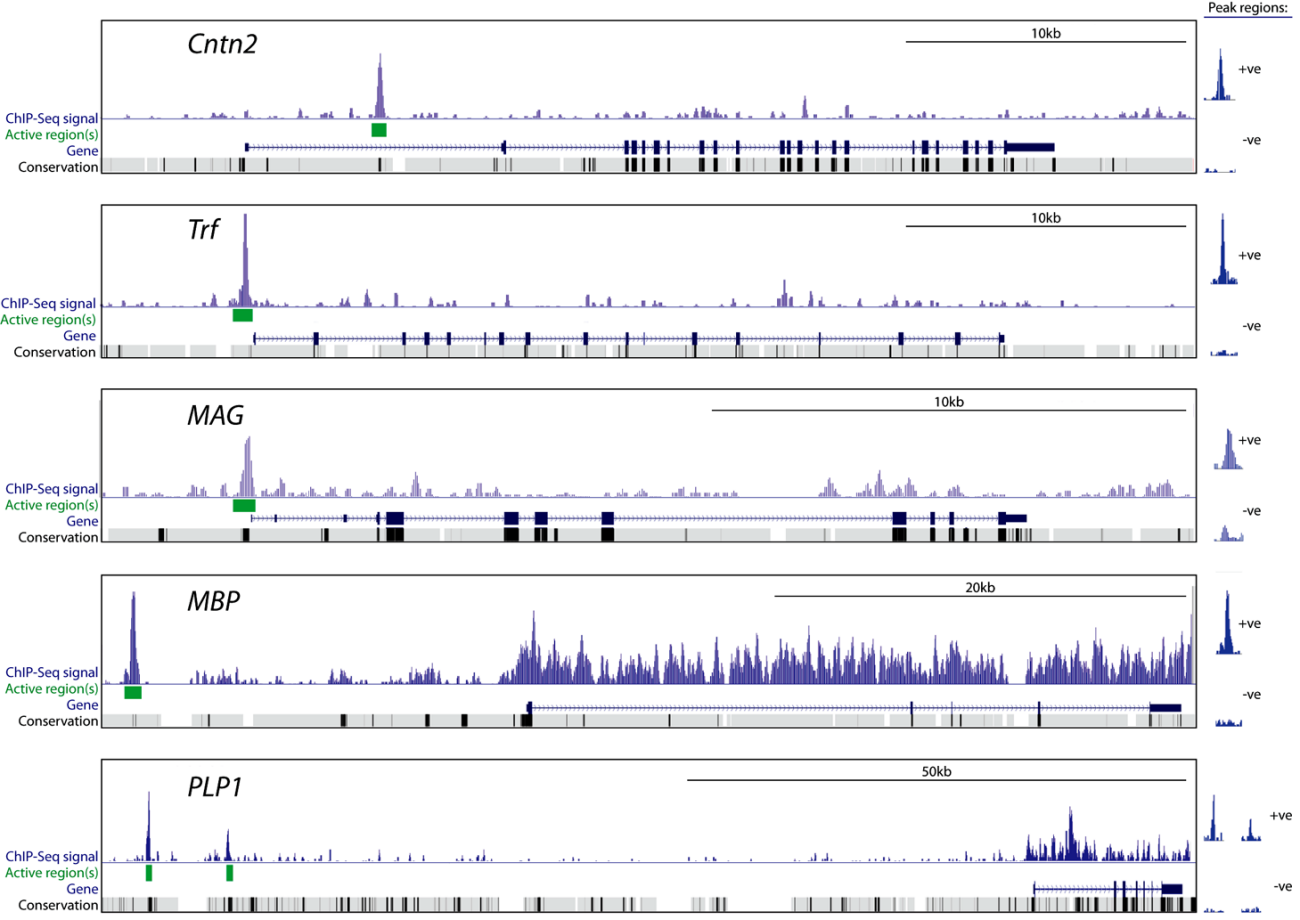
Examples of MYRF peaks proximal to oligodendrocyte-enriched genes. Signal track shows MYRF occupancy for the *Cntn2*, *Trf*, *Mag*, *Mbp*, and *Plp1* genes. Statistically significant peaks/active regions (green boxes) were identified in regions corresponding to conserved intronic regions (*Cntn2*), promoter regions (*Trf* and *Mag*), and upstream conserved regions (*Mbp* and *Plp1*). Inserts to the right show the Myc-MYRF signal and untagged MYRF control signal for each of the peaks/active regions identified. Note high background signal within the *Plp1* and *Mbp* genes (see text for further details).

For a more detailed analysis of the positioning of the MYRF peaks relative to TSSs, we plotted the incidence of MYRF peaks relative to the TSS of an unbiased gene list (17,090 rat refseq IDs) as well as the neuron-, astrocyte-, and oligodendrocyte-specific gene lists (Figure 3C). Plotting of MYRF peaks relative to the TSS of the unbiased gene list revealed a slight increase in MYRF binding in the 5 kb immediately upstream of the TSS, suggesting a modest overall relationship between MYRF binding and the TSS of genes in general. When we plotted MYRF binding relative to the TSSs of neuronal and astrocyte-specific genes, no relationship between the TSS and MYRF binding was evident. In contrast, there was a substantial increase in the incidence of MYRF binding around the TSS of the oligodendrocyte-specific genes. This enrichment was also evident for approximately 50 kb downstream of the TSS of the oligodendrocyte-specific gene list, again suggesting binding within intronic and downstream enhancers. MYRF occupancy at several well-established oligodendrocyte genes (*Plp1*, *Mbp*, *Mag*, *Trf*, and *Cntn2*), showing identified peaks for each, is shown in Figure 4. A full list of the 200 oligodendrocyte-specific genes and the location of the MYRF peaks relative to them is provided in Table S3.

Somewhat intriguingly, although the Chip-Seq background signal (“noise”) was low throughout most of the genome, high background was observed within a number of genes highly expressed by oligodendrocytes including *Plp1*, *Mbp*, *Cnp*, and the oligodendrocyte-enriched miRNA *miR219-2*. This background was present in both the Myc-MYRF and the untagged MYRF transfected samples (see Figure S2 for several examples), suggesting it did not reflect MYRF binding *per se*, nor was it identified as peaks by the MACS algorithm. The source of this background and its specificity to a small number of highly expressed transcripts is not clear, though it may be due to the “sono-seq” effect, in which open chromatin is preferentially fragmented by sonication and therefore overrepresented in sequencing data [27]. Although this background did not result in false-positive called peaks, it may have masked additional intronic MYRF binding sites within these highly expressed genes.

These findings indicate that the MYRF binding sites are not distributed randomly throughout the genome, but instead are overrepresented around genes usually induced during oligodendrocyte differentiation. Consistent with this, when we submitted the peak coordinates to GREAT (Genomic Regions Enrichment of Annotations Tool [28]), “axonal ensheathment” and “myelination” were the top two enriched gene ontology biological process terms for genes proximal to the MYRF peaks (*p* = 3.29e-6 and 5.50e-6, respectively; Table 1).

**Table 1:** Top five enriched gene ontology (GO) terms associated with MYRF ChIP-Seq peaks based on analysis with GREAT [28].

GO Biological Process	*p* Value	Observed Peak Hits	Observed Gene Hits	Total Genes in GO term
Axon ensheathment	3.29403e-6	32	23	63
Myelination	5.50192e-6	31	22	60
Stem cell development	3.86394e-5	36	20	63
Regulation of anti-apoptosis	1.44451e-4	27	17	45
Organ growth	2.38499e-4	21	14	32

### MYRF ChIP-Seq Peaks Identify Novel Enhancers of Myelin Gene Transcription

**Figure 5:**
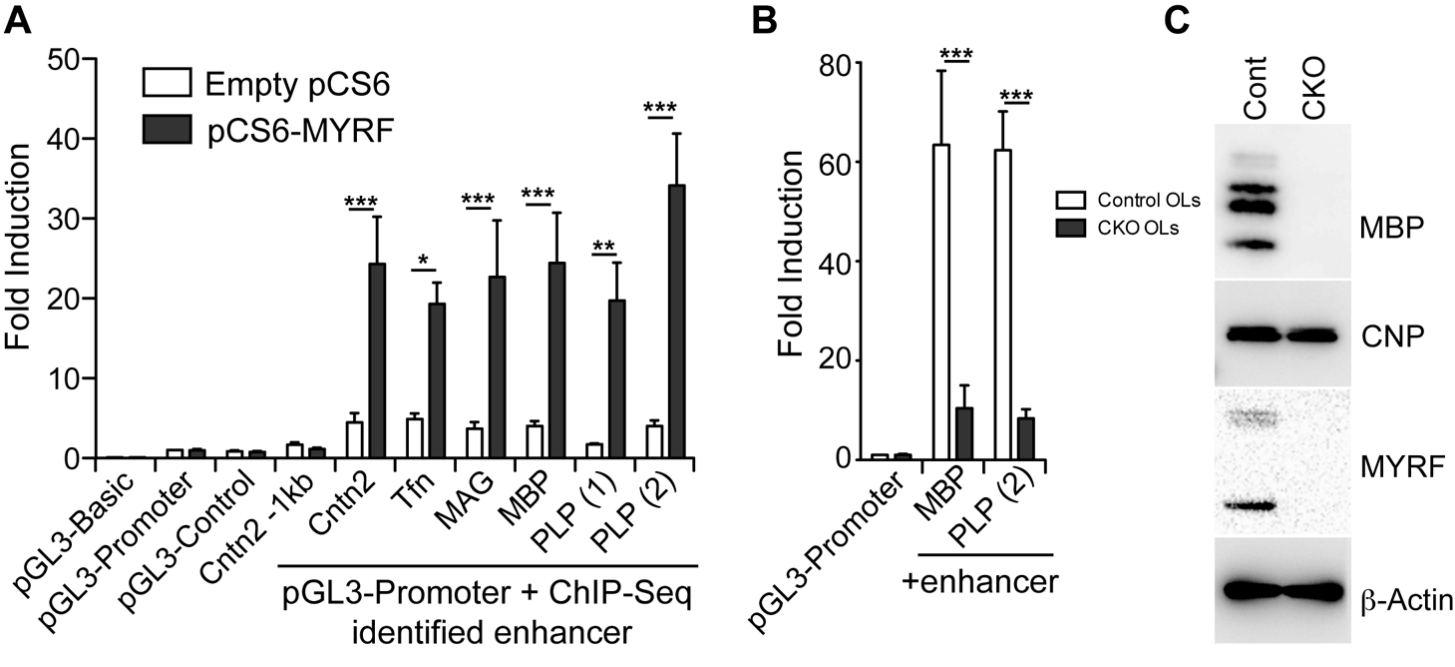
MYRF binding identifies enhancers of myelin gene expression. (A) DNA sequences (from 400–700 bp) encompassing the MYRF peaks proximal to the *Cntn2*, *Trf*, *Mag*, *Mbp*, and *Plp1* genes (Figure 4) were cloned into pGL3 upstream of the SV40 promoter and luciferase gene and co-transfected into the CG-4 cell line with either empty (pCS6) or MYRF overexpression (pCS6-MYRF) vectors. Co-expression of MYRF strongly induces luciferase expression in the presence of these enhancers, but has no effect on luciferase activity in their absence. (B) The MYRF-bound regions identified upstream of the *Mbp* and *Plp1* genes strongly promote luciferase expression in control oligodendrocytes relative to *Myrf* CKO oligodendrocytes, mirroring the loss of expression of MBP protein in the *Myrf* CKO cells (C). Fold inductions for all conditions are expressed relative to the pGL3-Promoter condition in control cells (pCS6 transfected in A, *Myrf* ^Wt/Fl^ in B). Data are shown as means and SEMs from 4–5 independent experiments. *p<0.05, **p = 0.01, **p<0.001 based on two-way ANOVA with Bonferroni posttest.

The positioning of the MYRF binding sites relative to oligodendrocyte-specific genes indicated that MYRF binding may identify *cis*-regulatory elements/enhancers for these genes. We therefore cloned a number of 400–700 bp DNA sequences encompassing the MYRF peaks shown in Figure 4 into the pGL3-promoter construct upstream of the SV40 promoter and the luciferase gene (see Table S4 for genomic coordinates of regions used and expression profiles of the associated genes). When transfected into CG-4 cells, these DNA regions modestly increased luciferase activity by several-fold relative to the control vector. Co-transfection of a MYRF expression vector with these putative enhancers induced luciferase expression by a further 4–12-fold. In contrast, MYRF co-expression had no effect on luciferase expression from the pGL3 vectors lacking enhancers (pGL3-Promoter), with an irrelevant SV40 enhancer (pGL3-Control) or with a control DNA region 1 kb upstream of the MYRF binding site identified within intron 1 of the *Cntn2* gene (Figure 5A).

To confirm that endogenous levels of MYRF can also regulate transcriptional activity from these elements, we selected two of these constructs, one from 19.1 kb upstream from the main *Mbp* TSS and one 80.7 kb upstream of the *Plp1* TSS, and transfected them into primary OPCs derived from either control (*Myrf^WT/FL^; Olig2^WT/Cre^*) or *Myrf* conditional knockout (*Myrf^Fl/FL^; Olig2^WT/Cre^*) mice. These OPCs were placed in differentiating conditions for 48 h before being assayed for luciferase activity to induce the expression of endogenous MYRF. The enhancers increased luciferase expression ∼60-fold in control oligodendrocytes relative to the promoter-only constructs, indicating that these ChIP-Seq-identified regions represent powerful oligodendrocyte enhancers. In contrast, only a modest increase in luciferase activity was seen in the MYRF conditional knockout cells (Figure 5B, *p*<0.001 between genotypes for each construct), mimicking the loss of endogenous MBP expression in these cells (Figure 5C).

### MYRF Binds DNA Via a Defined Consensus Sequence

**Figure 6:**
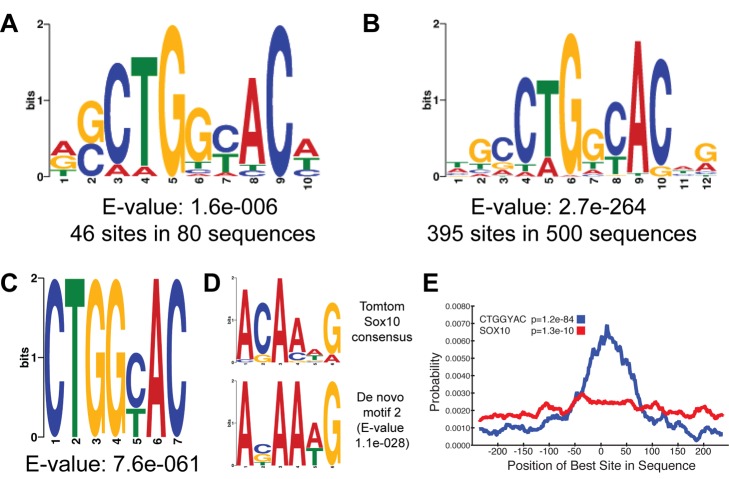
*De novo* identification of consensus sequences from MYRF peaks. (A) *De novo* sequence analysis using MEME from 80 peaks identified within 100 kb of oligodendrocyte-enriched genes identifies the sequence CTGGYAC, where Y = C or T. In separate analyses, essentially the same motif was identified using the 100 bp sequences surrounding the 500 strongest peaks using MEME (B) or 500 bp sequences of all 2,085 peaks using DREME (C). The second strongest motif identified in the DREME analysis from (C) was ACAA(A/T)G (D), a strong match for the known consensus sequence for Sox10. (E) Central enrichment analysis of the CTGGYAC and ANAA(A/T)G (Sox10) motifs in the 2,085 500 bp input sequences.

Previous work has shown that the putative DBD from the human ortholog *MYRF/C11Orf9* is not functionally interchangeable with the DBD of Ndt80, suggesting the MSE DNA consensus sequence recognized by the yeast members of the family may not be conserved throughout evolution [11]. To identify a DNA consensus motif for MYRF, we first submitted the central 100 bp sequences of 80 MYRF peaks proximal to oligodendrocyte-specific genes to MEME-ChIP [29] for *de novo* motif analysis. This analysis revealed a consensus sequence [G/C]CTGGYAC (where Y = C or T) as the strongest candidate (Figure 6A), which did not match any known consensus sequences for other transcription factors based on analysis with Tomtom [30]. This motif was confirmed in a broader *de novo* motif analysis using the central 100 bp from the 500 strongest peaks as input (Figure 6B), which also identified the 7 bp core CTGGYAC in 395 of the 500 sequences (E-value 2.7e-264). Parallel analysis submitting all 2,085 Myc-MYRF peak sequences to DREME [31] also yielded the same seven base pair motif with an E-value of 7.6e-061 (Figure 6C). Suggestively, the second most enriched motif in the DREME analysis was ACAA[A/T]G (E-value 1.1e-028), a close match to the consensus sequence for the oligodendrocyte transcription factor Sox10 (Figure 6D). Central enrichment analysis of the two motifs indicated that while the Sox10 motif showed little central enrichment within the input sequences, the CTGGYAC motif showed a strong central tendency (*p* = 1.2e-84), consistent with it being the primary binding motif for MYRF (Figure 6E).

**Figure 7:**
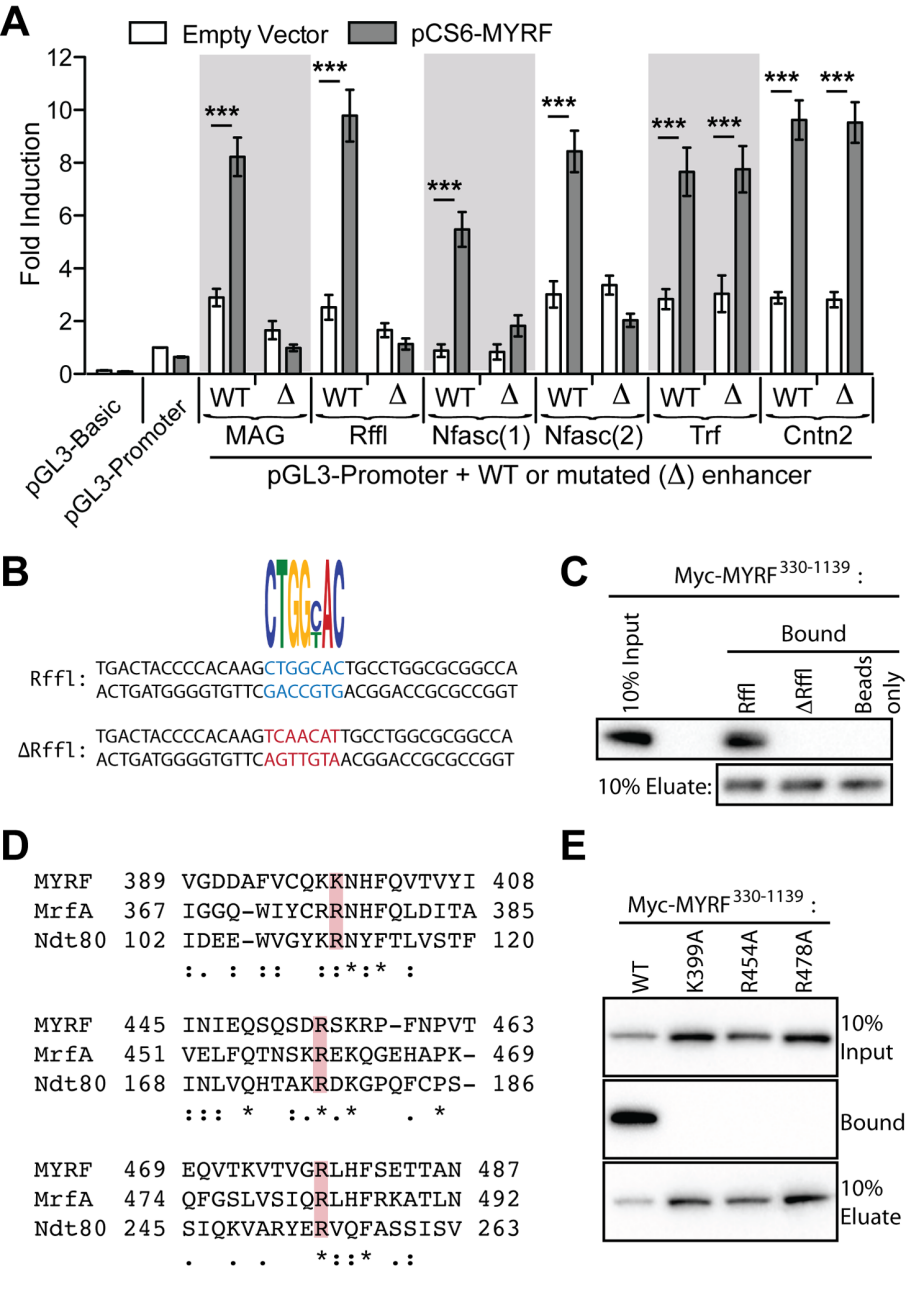
MYRF binds DNA via the CTGGYAC motif. (A) Mutational analysis of the CTGGYAC motif in six luciferase reporters containing MYRF-bound DNA regions from near the *Trf*, *Mag*, *Cntn2*, *Rffl*, and *Nfasc* genes in CG-4 cells. In four out of the six sequences, mutation of a single CTGGYAC sequence (Δ) was sufficient to abolish the effect of MYRF. Fold inductions for all conditions are expressed relative to the pGL3-Promoter and pCS6 co-transfected control cells. Data are shown as means and SEMs from three independent experiments. ***p<0.001. (B) DNA pulldowns using double-stranded oligonucleotides corresponding to the predicted MYRF binding site in the *Rffl* intronic enhancer, or equivalent oligonucleotides with the seven base pair motif mutated, conjugated to magnetic beads. The wild-type Rffl sequence efficiently captured Myc-MYRF^330–1139^ from cell lysates, whereas no interaction was detected for the mutated sequence or beads without DNA (C). (D) Sequence alignment between MYRF, *Dictyostelium* MrfA (Uniprot Q54PT9), and *S. cerevisiae* Ndt80 (Uniprot P38830) showing conservation of basic amino acids required for DNA binding by Ndt80 (highlighted). (E) DNA pulldown assay measuring interaction between the DNA sequence from the *Rffl* enhancer and the DNA binding domain of wild-type or mutant MYRFs. All detections performed with anti-Myc.

To assess the functional significance of the CTGGYAC motif, we selected six MYRF peaks with clear examples of the motif (the previously analyzed peaks from proximal to the *Trf*, *Mag*, and *Cntn2* genes, an intronic peak from the *Rffl* gene and two peaks from the first intron of the *Nfasc* gene, see Table S4) and assessed them in luciferase assays. In all six cases the wild-type sequences promoted luciferase expression when co-expressed with MYRF. In four cases (*Mag*, *Rffl*, and the two *Nfasc* peaks) PCR mutagenesis of the CTGGYAC motif completely abolished the effect of MYRF (Figure 7A). In the other two cases (*Cntn2* and *Trf*), mutation of the motif had no effect on the ability of MYRF to enhance transcription from these DNA regions, however other close matches for the CTGGYAC motif present in these enhancers may suggest redundancy in binding and explain the retained function.

To further confirm direct binding of MYRF to this motif, we performed a DNA pull-down assay, conjugating double-stranded 37 bp oligonucleotides, corresponding to the predicted binding site in the *Rffl* intronic enhancer, to magnetic beads (Figure 7B). These oligonucleotides could capture the DBD of MYRF from cell lysates. In contrast, corresponding oligonucleotides with the CTGGYAC motif mutated or beads alone showed no interaction with the DBD of MYRF (Figure 7C).

MYRF and its human ortholog (MYRF/C11Orf9) were initially identified as putative transcription factors due to apparent conservation of several basic amino acids required for the DNA binding activity of yeast Ndt80 [11],[12]. As Russel et al. [15] note, the overall degree of sequence homology between *Ndt80* and *MYRF* is quite low, however a recent report found that the key residues required for DNA binding by *Ndt80* are also required for DNA binding by *dictostelium* MrfA [32]. To assess whether this requirement is shared by the vertebrate orthologs, we made individual point mutants of the equivalent basic residues in MYRF (K339, R454, and R478; Figure 7D) and assessed their ability to interact with the *Rffl* intronic enhancer sequence (Figure 7B) in DNA pull-down assays. In agreement with their vital role for DNA binding in both yeast and *dictostelium*, mutation of each of the residues in MYRF led to a dramatic decrease in DNA binding. These DNA pull-down experiments demonstrate a bidirectional specificity of MYRF binding to DNA, requiring both conserved residues within the DNA binding domain of MYRF as well as a specific target DNA sequence.

### MYRF, Sox10, and Olig2 Display Overlapping Genomic Binding Sites

Given the overrepresentation of the Sox10 consensus motif in the regions bound by MYRF, as well as the similar CNS dysmyelination phenotype displayed in the absence of Sox10 and MYRF [8],[9],[13], it is tempting to speculate the two factors may target the same genes and/or regulatory elements. To assess this, we compared the MRF ChIP-Seq dataset with an independently generated Sox10 ChIP-Seq dataset generated from the developing rat spinal cord, in which 17,659 Sox10 peaks were identified (J. Svaren, unpublished data). Despite the independent generation of these datasets, there was a relatively high degree of direct overlap between the peaks for the two factors, with 30.3% of the MYRF peaks having an overlapping Sox10 peak. This overlap was particularly striking for regions near myelin genes. Nevertheless, in addition to the shared peaks, there were clear examples of peaks highly specific for each factor. These factor-specific peaks confirm specificity in the signal obtained for binding in each experiment, and also indicate the ability for each factor to bind independently of the other (see Figure S3 for examples surrounding the *Mobp*, *Mbp*, *Cntn2*, and *Josd2* genes). We extended this analysis by including a recently published dataset of 25,787 binding sites for the transcription factor Olig2 in differentiating rat oligodendrocytes [3]. There was also a significant degree of overlap between the binding sites for MYRF and Olig2 (40.2% of the MYRF peaks having an overlapping Olig2 peak). Overall, there were 517 sites of overlap between the peaks for all three transcription factors. This overlap points to a likely important functional relationship between these three factors in regulating key genes in the CNS myelination program.

## Discussion

We have previously demonstrated that *Myrf* is required for oligodendrocyte differentiation and the generation and maintenance of CNS myelin [13],[14]. Here, we uncover the molecular mechanisms by which MYRF acts to drive these processes. We find that MYRF is a transmembrane transcription factor that undergoes a proteolytic cleavage event to separate the N-terminal transcription factor from the transmembrane domain-containing C-terminal region. The N-terminal component of MYRF is targeted to the nucleus by at least one nuclear localization sequence and directly binds to the regulatory elements of genes involved in myelination to stimulate their transcription. The C-terminal cleavage product remains excluded from the nucleus. These results reconcile previous reports that propose transmembrane/endoplasmic reticulum localization and nontranscription factor function of the family [15],[16] and reports that have proposed a family of transcription factors defined by yeast *Ndt80* and including *MrfA* in *Dictyostelium* and *MYRF* in mice [11],[13],[32].

### MYRF Is a Novel Example of a Membrane-Associated Transcription Factor That Cleaves by a Unique Mechanism

There are varied examples of membrane-associated transcription factors in nature, including Notch, the SREBPs, and ATF6, which are synthesized as inactive membrane-bound precursors linked to the plasma membrane or endoplasmic reticulum. Upon a biological signal (binding of Jagged or Delta in the case of Notch, cholesterol in the case of the SREBPs and endoplasmic reticulum stress in the case of ATF6), these proteins are cleaved by Regulated Intramembrane Proteolysis (RIP) to release nuclear targeted products that effect transcription [18],[19],[33]. Like these factors, MYRF undergoes a cleavage event to release its DNA-binding N-terminal region from the transmembrane domain. Consistent with this, we found that overexpression of truncated MYRF constructs consisting of the proline-rich, DNA-binding, and CID domains (residues 1–763) but not the C-terminal region were sufficient to drive myelin gene expression (Figure S4).

In contrast to Notch, the SREBPs, and ATF6, however, the activating cleavage of MYRF occurs several hundred residues away from the transmembrane domain in a region homologous to the ICD of bacteriophage tailspike proteins. Mutation of key residues required for autoproteolytic cleavage of the tailspike proteins also blocks cleavage of MYRF, confirming that MYRF cleaves via this ICD rather than the RIP mechanism common to these previously described membrane-associated transcription factors. It is extremely tempting to speculate that the cleavage of MYRF is regulated in response to a biological signal, as is the case for other known membrane-associated transcription factors. To a large extent, however, MYRF's cleavage appears to be consitutive, as we observed cleavage when MYRF was expressed in cultured primary oligodendrocytes, the CG-4 oligodendrocyte cell line, 293T cells, or even when a GST-fusion construct including MYRF amino acids 330–765 was expressed in bacteria (Figure S1). Similarly, probing optic nerves with the anti-N-terminal and C-terminal antibodies at a series of ages ranging from postnatal day 8 to adult indicated at all stages the majority of MYRF protein in oligodendrocytes was present in the cleaved form, with the N-terminal antibody giving primarily nuclear staining. This is consistent with the function of the ICD in the bacteriophage endosialidase protein, which mediates autoproteolytic cleavage of the protein upon homotrimerization and proper folding [21]–[23]. As such MYRF's activating cleavage may simply occur following correct folding and self-association of the protein. Whether this is the case or whether it occurs in response to a biological signal will be an extremely important point to clarify in future work.

To our knowledge, within vertebrates this bacteriophage ICD is only represented by *MYRF*, the closely related paralog *Myelin Regulatory Factor-like/Gene Model 239*, and their orthologs. This cleavage mechanism appears to extend well back in evolution, however, with the DNA-binding portion of the MrfA protein in *Dictyostelium* having recently been reported to run as a ∼75 kda protein [32] and the serine/lysine dyad required for cleavage of the ICD also being present in the *Dictyostelium* protein and other orthologs (see Figure S5). The domain is not present in Ndt80, however, raising the intriguing possibility that the ICD may have arisen in bacteriophages and been transferred to eukaryotes via lateral gene transfer.

### MYRF Directly Targets Genes Underpinning Oligodendrocyte Differentiation and Myelination

Importantly, we demonstrate for the first time that MYRF directly regulates the expression of genes underlying myelination. Peaks in MYRF binding in the genome of primary oligodendrocytes were strongly overrepresented near oligodendrocyte-specific genes, many of which we have previously found to be reliant on MYRF for their expression. Luciferase assays confirmed that these MYRF-bound regions acted as strong MYRF-dependent enhancers of transcription. Due to the limitations of previous methods, studies looking at the direct targets of oligodendrocyte transcription factors have typically focused on activity at the promoters or nearby *cis* regulatory elements of well-defined myelin genes. For example, Sox10 and Olig1 have been shown to promote transcription at the *Mbp* promoter [9],[34]. Interestingly, we found that MYRF more commonly targets intronic or upstream enhancer regions than the direct promoter regions of target genes. Although we only focused on the 100 kb either side of the TSSs, enhancers can be located at distances of at least a megabase distal to target genes [35]. This represents a substantial challenge in definitively linking ChIP-Seq identified binding sites to their likely target genes.

In spite of this challenge, the use of ChIP-Seq methods in conjunction with genome-wide expression analysis offers an opportunity for broad target identification. MYRF binding sites were present proximal to genes encoding important protein components of myelin including *Mbp*, *Plp1*, *Mag*, and *Mog*. In addition, we were able to identify MYRF binding sites for genes with varied demonstrated roles in myelination/oligodendrocyte biology (see Table S3). These include genes encoding cytoskeletal proteins (*Tppp*, *Kif21a*), receptors (*Fgfr2*, *Gpr37*), oligodendrocyte/neuron junctional proteins (*Cntn2*, *Hapln2*, *Nfasc*), transmembrane proteins (*Kai1*, *Odz4*), lipid metabolism proteins (*Aspa*, *Elovl7*, *Fa2h*, *Ldrap1*, *Ugt8*, *Slc45a3*), as well as other transcription factors (*Sox10*, *Nkx6-2*, *St18*, *Smad7*). In addition, we were able to identify MYRF binding sites proximal to many oligodendrocyte-enriched genes whose roles in myelination have not yet been investigated, such as *Josd2*, *Rffl*, *Nipal4*, and *Rnf220*. These targets give a broader perspective of the cellular mechanisms by which MYRF coordinates the differentiation of oligodendrocytes and their myelination.

### MYRF Binds DNA Via a Defined Consensus Sequence

Based on the ChIP-Seq data, we were able to identify a novel seven base-pair consensus motif for MYRF (CTGGYAC). Mutation of this sequence in luciferase constructs was sufficient to prevent the activity of MYRF and prevent interaction with the DBD of MYRF in DNA pull-down assays. This motif is distinct from the binding motif for Ndt80 (CACAAA[A/G]) and also from recently described binding sequences for the *Dictyostelium* ortholog *MrfA* [32], suggesting that the binding preferences for this transcription factor family have diverged significantly during evolution. Although the CTGGYAC motif could be found near the centre of the majority of the strong MYRF peak sequences, it is unlikely to be the only determinant of MYRF binding to DNA. Intriguingly, the second most enriched motif identified in our ChIP-seq sequences was a good match for the consensus sequence of the oligodendrocyte transcription factor Sox10, and consistent with this, a strong degree of overlap was seen for ChIP-Seq peaks obtained for both MYRF and Sox10 (Figure S3). It seems likely that interactions between MYRF and Sox10 or other oligodendrocyte transcription factors as well as chromatin prepatterning by Olig2 and Brg1 [3] will play a large role in determining binding patterns and expression of key myelin genes.

Alignment of the MYRF protein with the ICD of the phage neck appendage protein (Figure 2A) revealed an overall 49.1% similarity within this region, with two sections showing notable amino acid identity. The first of these is the serine lysine dyad (serine 587 and lysine 592) that we demonstrate is required for MYRF cleavage. The second is the perfectly conserved GVIAQ sequence corresponding to the region required for trimerization of the phage proteins [21]. An implication of this is that MYRF, like the endosialidase proteins, may function as a homotrimer. This proposition is supported by a recent report that the *Dictyostelium* ortholog MrfA binds DNA via three distinct elements [32] and the findings of a co-submitted manuscript by Li and colleagues investigating the human MYRF/C11Orf9 protein. In our *de novo* DNA consensus sequence analysis, we found that when the MEME output parameters were set to exclude short motifs, the most enriched motif returned was a repeat of the CTGGYAC motif separated by 3 bp. Similarly, entering the CTGGYAC motif as both the primary and secondary motif in SpaMo (Spaced Motif Analysis Tool, [36]) found a significant paired incidence of the motif with 3 bp spacing within the 2,085 input sequences (*p*<0.001). This finding is consistent with MYRF binding DNA as a multimer, though the majority of ChIP-Seq-identified binding sites had a single CTGGYAC motif, suggesting that a single copy is adequate for biologically relevant levels of binding. The exact stoichiometry of optimal binding sites and how this relates to MYRF's regulation of gene expression will be an important point to clarify in future work.

### How Is MYRF Regulated During Oligodendrocyte Differentiation?

This study greatly clarifies the molecular mechanisms by which MYRF promotes myelination, demonstrating that it directly binds to and promotes the expression of genes underpinning the myelination process. A number of important questions remain for future studies. Firstly, what is the role of the C-terminal portion of MYRF? Is its role simply to ensure the correct folding, self-association, and cleavage of the protein, or does it have additional binding partners and regulatory roles that influence the function of MYRF in myelination? Secondly, the consensus sequence for Sox10 was overrepresented in regions targeted by MYRF, indicating these two factors likely interact in their regulation of target genes. Consistent with this, mutations for each gene result in a block in oligodendrocyte differentiation at the premyelinating stage [8],[13]. Clarification of the functional relationship between these factors will be important to clarify. Finally, although the expression of MYRF is strongly induced during oligodendrocyte differentiation, the signals regulating this induction are essentially unknown. Recent reports indicate that its expression may be influenced by the coordinated activity of *Olig2* and *Brg1* [3] and by posttranscriptional regulation by miRNAs [37]. Elucidation of the upstream pathways and signals that induce MYRF will be important both for understanding the molecular control of the myelination program, but also potentially for identifying strategies to promote remyelination in demyelinating disease.

## Materials and Methods

### Animal Work

Generation and use of the *Myrf^Floxed^* and *Olig2^Cre^* mouse lines to generate *Myrf* CKO oligodendrocytes have previously been described [13],[38]. All experiments were approved by and conducted in accordance with the Florey Institute of Neuroscience and Mental Health Animal Ethics committee.

### Cell Culture

Primary OPCs were isolated from enzymatically dissociated brains as previously described [10]. Briefly, mouse OPCs were positively immunopanned from dissociated *Myrf^Wt/Fl^*; *Olig2^Wt/Cre^* or *Myrf^Fl/Fl^; Olig2^Wt/Cre^* cortices using anti-PRGFRα (BD Pharmingen, Cat. No. 558774) after removal of contaminant microglia by panning with BSL1 (Abacus ALS, Cat. No. l-1100). Rat OPCs were positively immunopanned with anti-O4 after removal of astrocytes and postmitotic oligodendrocytes with anti-Ran-2 and anti-GalC, respectively. Cells were grown in SATO serum-free media as previously described [39], with the addition of 2% SM1 neuronal supplement (Stemcell Technologies, Cat. No. 05711) for mouse cells. PDGF-AA (10 ng/ml, PeproTech), NT-3 (1 ng/ml, PeproTech), and CNTF (10 ng/ml, PeproTech) were added to the media to proliferate OPCs; PDGF-AA was removed from the media for 48 h to stimulate differentiation into postmitotic (PDGFRα−, MBP+) oligodendrocytes. The CG-4 cell line [20] was maintained in the same conditions as rat OPCs. 293T cells were maintained in DMEM with 10% FCS, 1% Pen-Strep, 2 mM glutamine, and 1 mM sodium pyruvate. The CG-4 and 293T cell lines were transfected using Effectene (Qiagen) as per the manufacturer's instructions. Primary cells were transfected using the Amaxa nucleofection system as previously described [13]. Cells were analyzed by immunofluorescence, Western blot, or luciferase assay 48 h after transfection unless otherwise stated.

### ChIP-Seq

Cultured primary rat OPCs (20×10^6^ per condition) were transfected with either pCMV-Sport6-Myc-MYRF or pCMV-Sport6-MYRF. Cells were then cultured for 48 h in differentiative conditions (−PDGF, +40 ng/ml triiodothyronine; Sigma) to allow time for construct expression and for the cells to differentiate to the point where MYRF would usually be endogenously expressed and active. Chromatin-immunoprecipitation against the Myc-tag (Abcam ab9132) was performed on formaldehyde cross-linked chromatin (∼50 µg/sample) by GENpathway (San Diego, California), as were subsequent analyses. Briefly, ChIP samples were used for Illumina library construction and libraries were submitted to the Stanford Functional Genomics Facility for single-read 36-base Illumina sequencing (>20 million reads/sample). The 35-nt sequence reads were mapped to the rat 2004 genome (rn4) assembly using the ELAND algorithm, using only tags that mapped uniquely with no more than two mismatches. Sequence tags were 3′ extended *in silico* to 110 bp. BAR files were generated based on mapping fragments to the genome in 32 nucleotide bins and were viewed in the Affymetrix Integrated Genome Browser (IGB). Peaks/intervals were identified using the MACS peak finding algorithm [40] comparing Myc-MYRF ChIP results against control MYRF ChIP results, using a moderate to low cutoff (*p* value = 10exp-6) and a threshold of 16 tags. Based on the number of negative peaks, FDR was estimated to be 12.3%. Browser Extensible Data (BED) files of the interval and peak coordinates were compiled for viewing the data on the UCSC browser [41].

### Subsequent Analysis of ChIP-Seq Data

For analysis of peak proximity to cell-type specific genes, the top ranked 200 mouse genes for each cell type [10] were converted to the RatRefseq reference gene orthologs using BioDBnet [42]. For each transcript cluster ID on the exon array, a genbank nucleotide accession number obtained from the MoEx-1_0-st-v1.na32.mm9 Affymetrix annotation file from Netaffx [43] was submitted to BioDBnet. BED files of gene lists for each cell type with genomic locations for each rat gene ID and a BED file of genomic coordinates or all Rat refseq genes were obtained from the UCSC table browser [41]. Mouse genes that did not return a rat Refseq ID were manually curated and if a Rat gene model corresponding to the mouse gene could be identified, its corresponding genomic location was manually annotated. In a few cases, we used mouse gene IDs where no appropriate Rat gene ID was available. Python (2.7.2) custom scripts were used in an analysis pipeline with intersectBed from Bedtools version 2.16.2 [44] to identify all MYRF binding peaks within a region 100 kb upstream and 100 kb downstream from the TSS. Only Refseq genes with unique TSS were included in the final analysis so as not to overrepresent genes with multiple transcripts. The results were plotted using matplotlib [45]. For *de novo* motif analysis, 100 bp or 500 bp sequences were taken flanking the identified Myc-MYRF peaks and submitted to the MEME-ChIP suit (http://meme.nbcr.net/meme/), which incorporates the MEME (Multiple Em for Motif Elicitation), DREME, and CentriMo programs [29]. For analysis using the Genomic Regions Enrichment of Annotations Tool [28], the rat ChIP-Seq peak coordinates were converted to the mouse NCBI37/mm9 genomic assembly using the UCSC browser liftover function and the coordinates submitted to GREAT using a 50 kb regulatory region setting. Overlap analysis for MYRF, Sox10, and Olig2 peaks was performed using the intersect function of the UCSC browser.

### *In Silico* Analysis of MYRF Features

The UniProt mouse MYRF sequence (accession number Q3UR85) was submitted to several subcellular targeting motif and feature predictors including ELM [46], PSORTII [47], NucPred [48], TMPRED [49], HMMTOP [50], Das-TMfilter [51], Toppred II [52], Paircoil2 [53], and to the protein databases PFAM [54] and Prosite [55]. HHPRED [56] and the NCBI PSI-BLAST and conserved domain BLAST tool [57] were used to identify the ICD, using residues 546–763 as input. Protein alignments were performed using ClustalW2.

### Luciferase Assays

Luciferase assays were performed using the Promega dual luciferase reporter assay kit as per the manufacturer's instructions. All cells were co-transfected with both the test pGL3 luciferase construct and a constitutive renilla construct, and luciferase readings normalized to the renilla levels. All luciferase results are shown as the mean and SEM of at least three independent experiments, with two-way ANOVA with Bonferroni posttests used to calculate statistical significance. Table S4 provides a list of the genomic regions cloned into the pGL3 promoter as putative enhancers, as well as the oligodendrocyte-specific genes with which they are associated.

### PCR Mutagenesis

Site-directed mutagenesis was performed using the Thermo Scientific Phusion High-Fidelity PCR Kit and primers as indicated in Table S5. Forward and reverse primers were used in separate reactions with 50 ng template DNA. After an initial denaturation at 98°C for 45 s, reactions were run in 10 cycles of 15 s denaturation at 98°C and 6 min combined annealing and extension at 72°C. Following combination of 25 µl forward and reverse reactions and addition of 0.75 µl fresh Phusion DNA Polymerase, another initial denaturation and 18 cycles as described above were performed. Methylated template DNA was then digested by incubation with DpnI, products purified using a QIAquick PCR Purification Kit (Qiagen), and used to transform OneShot Top10 chemically competent bacteria (Invitrogen). Single colony clones were verified via DNA sequencing.

### DNA Pulldown Assay

For DNA pulldown assays CG-4 cells were transfected with the MRF Myc-DNA-C constructs (Myc-tagged residues 328–1139 or respective mutants for that construct) for 24 h before being lysed in NP40 buffer (150 mM NaCl, 1% Nonidet P-40, 50 mM Tris-Cl, 1 mM PMSF). Lysates were clarified by centrifugation at 15,000× *g* for 20 min at 4°C and adjusted to a total protein concentration of 1 µg/µl. To prepare oligonucleotide-conjugated beads, 30 µl Dynabeads MyOne Steptavidin T1 (Invitrogen) were washed three times in 500 µl buffer A (5 mM Tris pH 8.0, 0.5 mM EDTA, 1 M NaCl) and then incubated in 100 µl buffer A containing 1 µg biotinylated, annealed oligonucleotides for 30 min at room temperature with rotation. The oligonucleotide-conjugated beads were washed twice with 500 µl buffer A, three times with 500 µl buffer C (20 mM Tris pH 8.0, 1 mM EDTA, 10% glycerol, 1 mM DTT, 50 mM NaCl) before being resuspended in 100 µl buffer C and added to 300 µl cell lysate and sheared salmon sperm (final concentration 0.2 µg/µl). Beads were incubated with cell lysates for 30 min at room temperature with rotation and then washed three times in 500 µl buffer C. Bound protein was eluted boiling the beads in 2× Laemmli buffer and subject to gel electophoresis and detection by Western blot with anti-Myc (clone A46; Merck Millipore). Forty µl of pre-bead cell lysate and 40 µl of nonbound protein from the beads served as 10% input and eluate controls, respectively.

### Bacterial Expression of GST-Fusion Constructs

MYRF constructs encoding residues 393–540 or 393–766 were ligated into pGEX-6P-3 vector (GE Healthcare) in frame with GST and transformed into the BL21(DE3)pLysS-T1^R^ stain (Sigma). Expression was induced by addition of 0.2 mM IPTG for 2 h at 28°C before bacteria were lysed and the fusion proteins column purified using glutathione Sepharose 4B (GE Healthcare). The eluted GST-MYRF^393–766^ protein was used to generate monoclonal antibodies (Walter and Eliza Hall Institute monoclonal facilities), also confirming the presence of the cleavage in bacterial expression systems (Figure S1).

### Immunostaining

Mice for immunohistochemistry were anesthetized with sodium pentobarbital (100 mg/kg, i.p.) and perfused with PBS and 4% PFA. Tissue was harvested and postfixed for 2 h in 4% PFA before being sunk in 30% sucrose and processed for cryosections. Cells for immunostaining were fixed for 10 min in 4% PFA and washed in PBS. For immunostaining, cells on coverslips or cryosections were blocked for 1 h in 10% normal goat serum and 0.3% Triton X-100 in PBS and incubated overnight in primary antibodies diluted in blocking solution. Coverslips or cryosections were then washed three times in PBS and stained with appropriate fluorescent conjugated antibodies (Alexa Fluor; Life Technologies) for 1 h, washed three times in PBS, and coverslipped. Primary antibodies were anti-N-terminal MYRF (1∶10,000, Wegner lab internal polyclonal, raised against the N-terminus of MYRF), anti-C-terminal MYRF mAb (1∶500, Emery lab internal monoclonal raised against GST-MYRF^393–766^ and mapped to the C-terminal fragment), anti-Myc tag (1∶500, Millipore 4A6), rabbit anti-Flag (1∶500, Cell signaling #2368), mouse anti-Flag (1∶500, Sigma F3165), mouse anti-gm130 (1∶100, BD Biosciences #610822), rabbit anti-calnexin (1∶500, Sigma C4731), CC1 monoclonal (1∶500, Calbiochem OP80), rabbit anti-Sox10 (1∶500, Millipore AB6727), and chick anti-GFP (1∶20,000 Abcam ab13970).

## Supporting Information

Figure S1 **Posttranslational cleavage of MYRF maps to shortly after the DBD in both mammalian and bacterial cells.** (A) Western blot analysis of N-terminal Myc-tagged truncated MYRF constructs expressed in 293T cells. All constructs that include the proline-rich region, DBD, and up to or including the transmembrane region (Myc-MYRF, Myc-Proline-TM−, and Myc-Proline-TM+) give the same 75 kDa N-terminal cleavage product. The Myc-Proline-DBD construct (including resides 2–540 of MYRF) gives a product several kDa smaller. Similarly, the truncated Myc-DBD-C construct (residues 328–1139) product runs several kDa larger than the Myc-DBD construct (residues 328–540). Anti-Actin and anti-GFP are used to confirm protein loading and transfection levels, respectively. (B) Expression of GST-fusion constructs including residues 393–540 of MYRF (the DBD; predicted size 52.5 kDa) or residues 393–766 (the DBD and up to the transmembrane domain; predicted size 76.3 kDa). As in mammalian cells, the bacterially expressed MYRF fusion construct is subject to cleavage shortly after the DBD. Both constructs are expressed in the BL21 (DE3) pLysS-T1^R^ *E. coli* strain at 28°C, purified 2 h after IPTG induction, and visualized with coomassie blue on a denaturing gel.
(TIF)

Figure S2 **ChIP-Seq “noise” within transcribed regions of highly expressed oligodendrocyte genes.** ChIP-Seq signal from the Myc-MYRF sample and the untagged control sample (MYRF) for the genomic regions surrounding the *Mbp* (chr18), *Plp1* (chrX), *mir219-2* (chr3), and *Cldn11* (chr10) genes. Note the presence of MACS-identified peaks corresponding to specific signal within the Myc-MYRF sample (*) as well as the increased background signal present in both the Myc-MYRF and the untagged control samples within the transcribed regions of the genes. This high background was only observed within a small number of genes highly expressed by oligodendrocytes.
(TIF)

Figure S3 **Comparison between peaks for MYRF, Sox10, and Olig2.** (A–D) ChIP-Seq signal for MYRF in cultured oligodendrocytes and Sox10 in the rat spinal cord for the genomic regions surrounding the oligodendrocyte-enriched *Cntn2* (chr13), *Mobp* (chr8), *Mbp* (chr18), and *Josd2* (chr1) genes. Peaks were identified that were specific to MYRF (e.g., in intron 1 of the *Cntn2* gene) or specific to Sox10 (e.g., in the *Mobp* promoter and several kb upstream of the *Mbp* TSS) as well as peaks shared by both factors (e.g., the peak 19.1 kb upstream of the *MBP* TSS and one downstream of the *Josd2* gene). Note consensus sequences for both MYRF and Sox10 in this shared *Josd2* peak. (E) Analysis of the degree of direct overlap between peaks obtained for MYRF and Olig2 [3] in differentiating oligodendrocytes and Sox10 in the spinal cord. * denotes identified peak.
(TIF)

Figure S4 **Expression of the N-terminal region of MYRF is sufficient to promote myelin gene expression.** (A) Luciferase assay co-expressing Myc-tagged truncated MYRF constructs with the pGL3-Plp1 (2) construct (see Figure 5) in the CG-4 cell line. Expression of either the full-length MYRF or any construct containing both the proline-rich and Ntd80/DBD is sufficient to promote luciferase expression from the −80.7 kb *Plp1* enhancer and sv40 promoter. All fold-inductions are relative to pGL3-promoter and non-MYRF transfected cells, and data are shown as means and SEMs from three independent experiments. (B) Primary rat oligodendrocytes were co-transfected with GFP and the Myc-tagged truncated MYRF constructs, seeded in proliferative conditions (+PDGF), and assayed for MBP expression 72 h posttransfection (four coverslips per condition). Expression of either the full-length construct or constructs including up to or including the transmembrane region promoted MBP expression. (C) Schematic of the protein domains included in each construct used in (A) and (B). One construct consisting of the proline-rich and DBDs only (residues 2–540) was sufficient to drive luciferase expression in luciferase assays (A) but not MBP expression in primary cells (B). Statistical significance calculated via one-way ANOVA with Bonferroni posttest, comparing all conditions to the empty vector control. *p<0.05, **p<0.01, **p<0.001, ****p<0.0001.
(TIF)

Figure S5 **Conservation of the ICD region.** Alignment of the ICD for human MYRF/C11Orf9 protein and its orthologs, as well as bacteriophage proteins GA-1 neck appendage protein and Endo-N-acetylneuraminidase, showing conservation of the serine lysine dyad required for cleavage (first and fifth residues in the alignments).
(TIF)

Supporting Information S1 **Peak coordinates.** Genomic coordinates of MyRF (-ve control) and Myc-Myrf ChIP-Seq peaks. Coordinates are based on the *Rattus norvegicus* genome Rn4.
(XLSX)

Table S1 **Programs used to predict MYRF protein features.** Online programs used to identify the predicted features of the MYRF protein and the associated E-values or scores for each feature are listed. *Values represent E-values unless otherwise stated as being a *p* value or a score. ^†^Using MYRF residues 546–763 as input.
(DOCX)

Table S2 **Gene lists used to assess MYRF binding proximal to neuron-, astrocyte-, or oligodendrocyte-specific genes.** Cell-type-specific gene lists were generated from [10] using the Affymetrix All-Exon dataset (Mouse Exon 1.0 ST array). Where a mouse gene from the list could not be reliably mapped to the rat genome, it was omitted from the list and the next most enriched gene used.
(XLSX)

Table S3 **Position of MYRF peaks relative to the TSSs of oligodendrocyte-specific genes.** Table giving the transcript IDs and genomic coordinates of the TSS for each of the 200 oligodendrocyte-specific genes, as well as the positions of any MYRF peaks detected within 100 kb of these TSSs. Links are provided to the expression data for each gene.
(XLSX)

Table S4 **Genomic coordinates of genomic regions used for luciferase assays.** Genomic coordinates of the regions of the genome (typically ∼700 bp) corresponding to MYRF peaks that were cloned into pGL3-Promoter vector for luciferase assays. The left columns show the genes that these MYRF peaks are associated with and the genes' expression levels in CNS cell types ([10], using the Affymetrix All-Exon dataset). The right column shows the ChIP-Seq signal within each region cloned into pGL3-Promoter (note that the peaks are not necessarily centered in the amplified region as availability of acceptable primer sites was a consideration in determining regions to be cloned).
(DOCX)

Table S5 **Sequences of mutagenesis primers.**
(DOCX)

## Abbreviations

CNS: central nervous system
ChIP-Seq: chromatin-immunoprecipitation sequencing
Cntn2: Contactin 2
DBD: DNA binding domain
ICD: intramolecular chaperone domain
MAG: myelin associated glycoprotein
MBP: myelin basic protein
MOG: myelin oligodendrocyte glycoprotein
MYRF: myelin regulatory factor
Nfasc: neurofascin
NLS: nuclear localization signal
PLP1: Proteolipid Protein 1
Rffl: ring finger and FYVE-like domain containing
RIP: regulated intramembrane proteolysis
SREBP: sterol regulatory element binding protein
Trf: transferrin
TSS: transcription start site
