# Supplementary figures and images for: MYRF Is a Membrane-Associated Transcription Factor That Autoproteolytically Cleaves to Directly Activate Myelin Genes
Source: PLoS Biol. 2013 Aug 13;11(8):e1001625. doi: 10.1371/journal.pbio.1001625 (PMC3742440; doi:10.1371/journal.pbio.1001625)

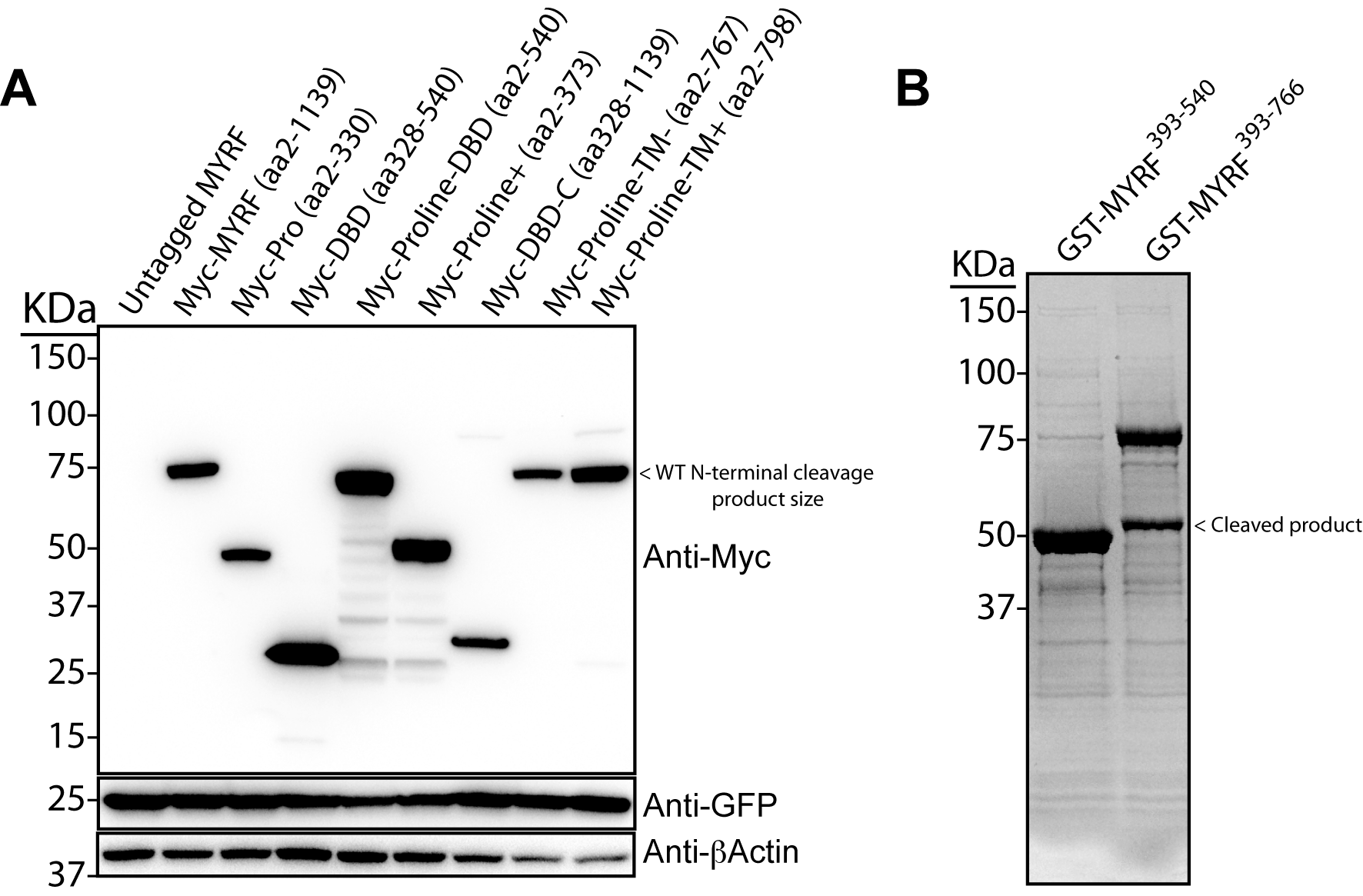

Supplement: Figure S1 — Posttranslational cleavage of MYRF maps to shortly after the DBD in both mammalian and bacterial cells. (A) Western blot analysis of N-terminal Myc-tagged truncated MYRF constructs expressed in 293T cells. All constructs that include the proline-rich region, DBD, and up to or including the transmembrane region (Myc-MYRF, Myc-Proline-TM−, and Myc-Proline-TM+) give the same 75 kDa N-terminal cleavage product. The Myc-Proline-DBD construct (including resides 2–540 of MYRF) gives a product several kDa smaller. Similarly, the truncated Myc-DBD-C construct (residues 328–1139) product runs several kDa larger than the Myc-DBD construct (residues 328–540). Anti-Actin and anti-GFP are used to confirm protein loading and transfection levels, respectively. (B) Expression of GST-fusion constructs including residues 393–540 of MYRF (the DBD; predicted size 52.5 kDa) or residues 393–766 (the DBD and up to the transmembrane domain; predicted size 76.3 kDa). As in mammalian cells, the bacterially expressed MYRF fusion construct is subject to cleavage shortly after the DBD. Both constructs are expressed in the BL21 (DE3) pLysS-T1R E. coli strain at 28°C, purified 2 h after IPTG induction, and visualized with coomassie blue on a denaturing gel. (TIF) [file pbio.1001625.s001.tif]

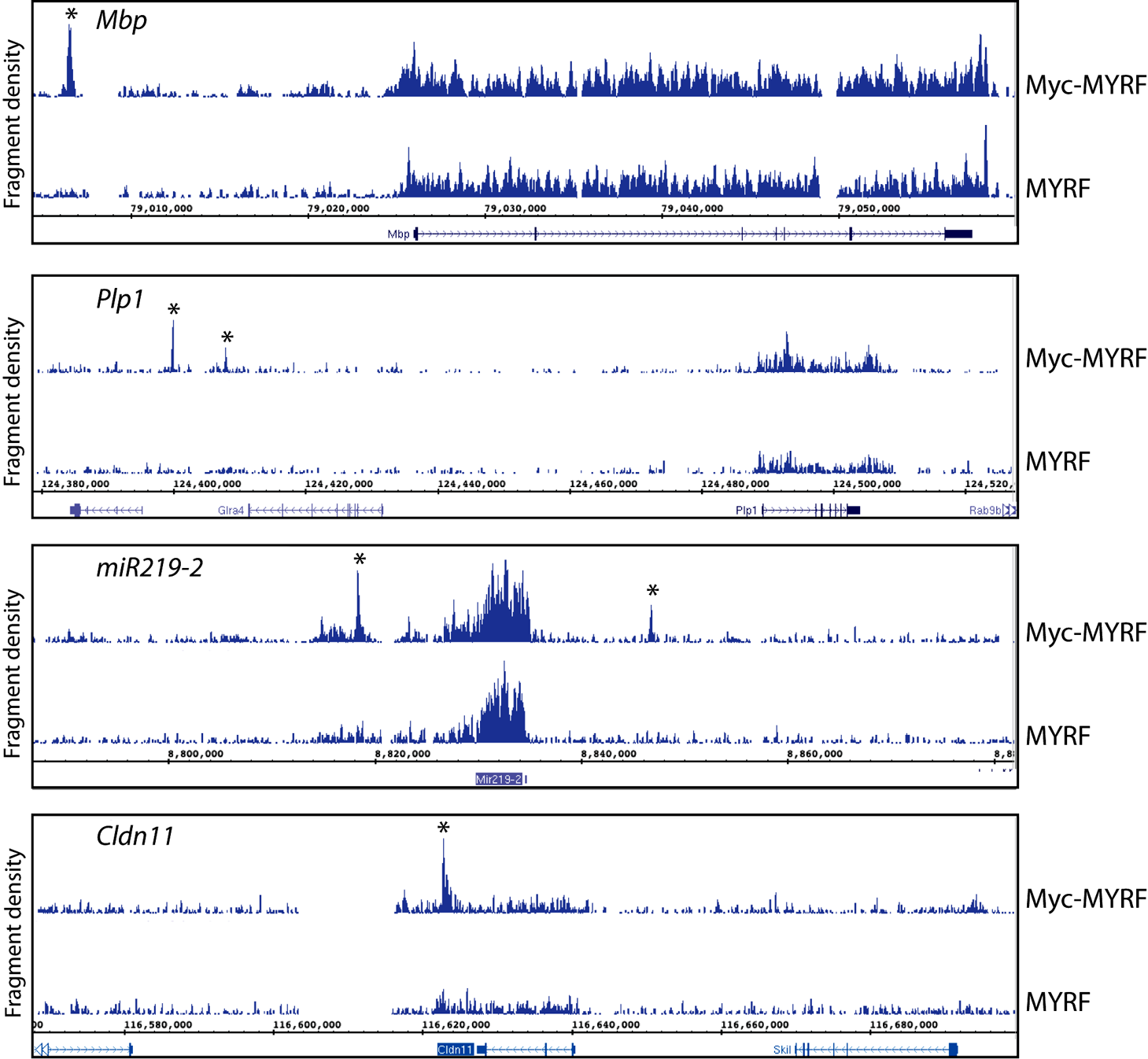

Supplement: Figure S2 — ChIP-Seq “noise” within transcribed regions of highly expressed oligodendrocyte genes. ChIP-Seq signal from the Myc-MYRF sample and the untagged control sample (MYRF) for the genomic regions surrounding the Mbp (chr18), Plp1 (chrX), mir219-2 (chr3), and Cldn11 (chr10) genes. Note the presence of MACS-identified peaks corresponding to specific signal within the Myc-MYRF sample (*) as well as the increased background signal present in both the Myc-MYRF and the untagged control samples within the transcribed regions of the genes. This high background was only observed within a small number of genes highly expressed by oligodendrocytes. (TIF) [file pbio.1001625.s002.tif]

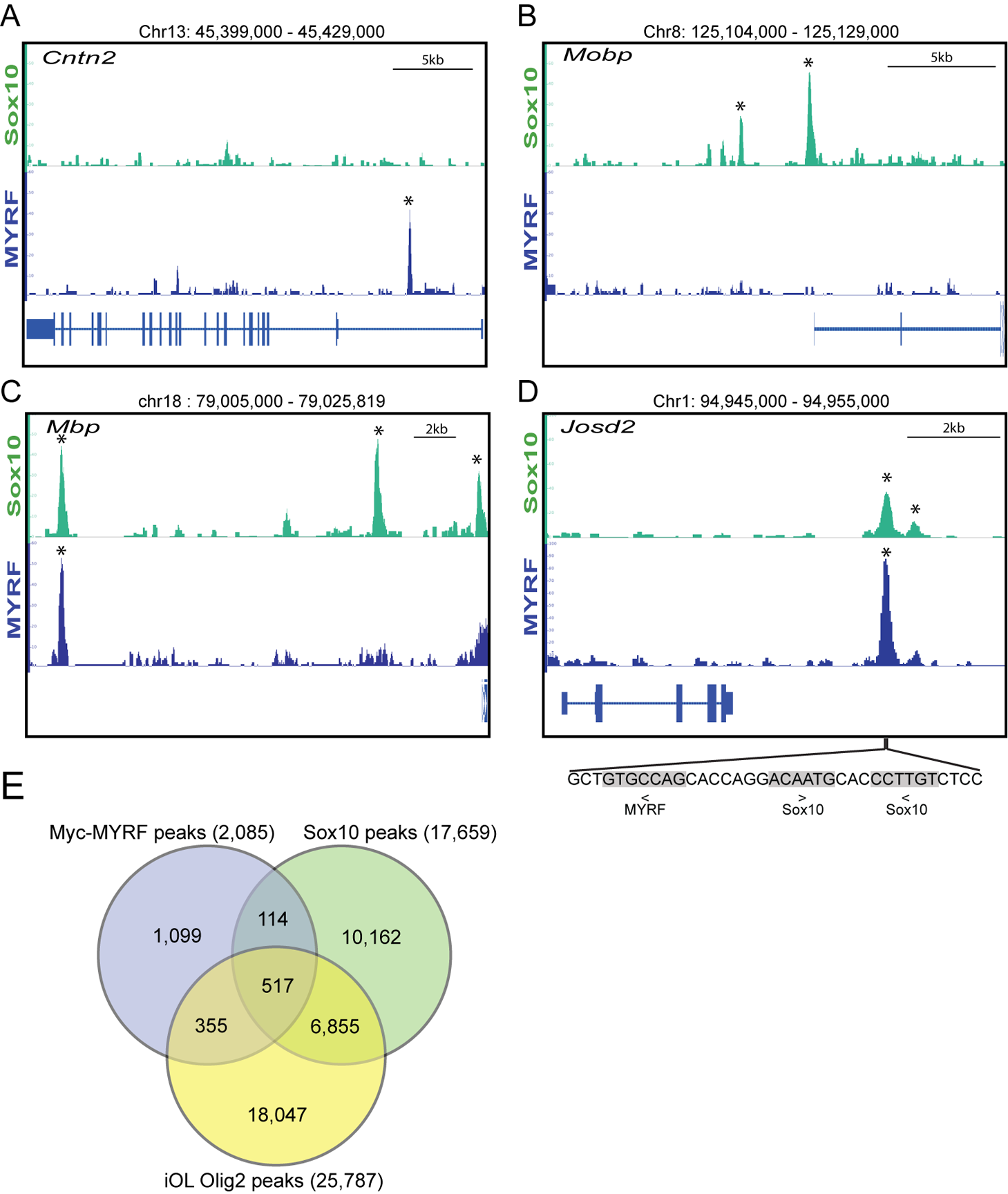

Supplement: Figure S3 — Comparison between peaks for MYRF, Sox10, and Olig2. (A–D) ChIP-Seq signal for MYRF in cultured oligodendrocytes and Sox10 in the rat spinal cord for the genomic regions surrounding the oligodendrocyte-enriched Cntn2 (chr13), Mobp (chr8), Mbp (chr18), and Josd2 (chr1) genes. Peaks were identified that were specific to MYRF (e.g., in intron 1 of the Cntn2 gene) or specific to Sox10 (e.g., in the Mobp promoter and several kb upstream of the Mbp TSS) as well as peaks shared by both factors (e.g., the peak 19.1 kb upstream of the MBP TSS and one downstream of the Josd2 gene). Note consensus sequences for both MYRF and Sox10 in this shared Josd2 peak. (E) Analysis of the degree of direct overlap between peaks obtained for MYRF and Olig2 [3] in differentiating oligodendrocytes and Sox10 in the spinal cord. * denotes identified peak. (TIF) [file pbio.1001625.s003.tif]

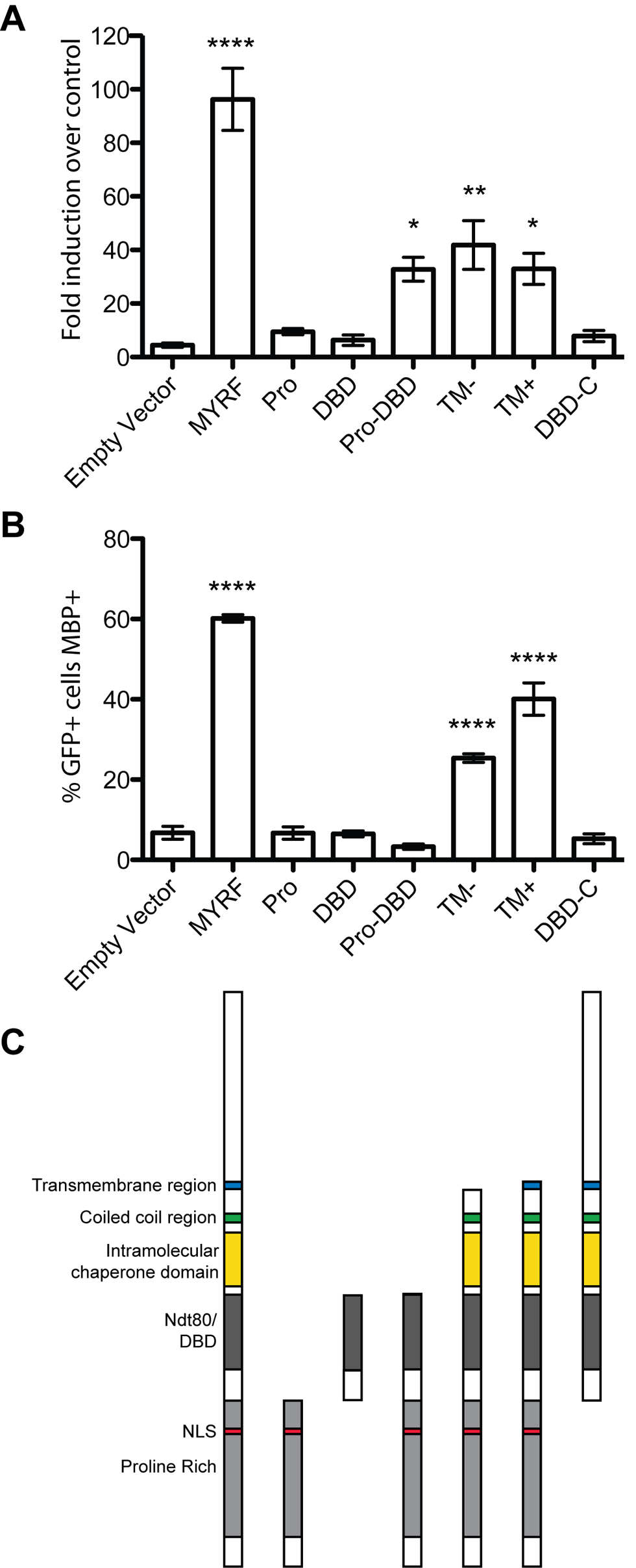

Supplement: Figure S4 — Expression of the N-terminal region of MYRF is sufficient to promote myelin gene expression. (A) Luciferase assay co-expressing Myc-tagged truncated MYRF constructs with the pGL3-Plp1 (2) construct (see Figure 5) in the CG-4 cell line. Expression of either the full-length MYRF or any construct containing both the proline-rich and Ntd80/DBD is sufficient to promote luciferase expression from the −80.7 kb Plp1 enhancer and sv40 promoter. All fold-inductions are relative to pGL3-promoter and non-MYRF transfected cells, and data are shown as means and SEMs from three independent experiments. (B) Primary rat oligodendrocytes were co-transfected with GFP and the Myc-tagged truncated MYRF constructs, seeded in proliferative conditions (+PDGF), and assayed for MBP expression 72 h posttransfection (four coverslips per condition). Expression of either the full-length construct or constructs including up to or including the transmembrane region promoted MBP expression. (C) Schematic of the protein domains included in each construct used in (A) and (B). One construct consisting of the proline-rich and DBDs only (residues 2–540) was sufficient to drive luciferase expression in luciferase assays (A) but not MBP expression in primary cells (B). Statistical significance calculated via one-way ANOVA with Bonferroni posttest, comparing all conditions to the empty vector control. *p<0.05, **p<0.01, **p<0.001, ****p<0.0001. (TIF) [file pbio.1001625.s004.tif]

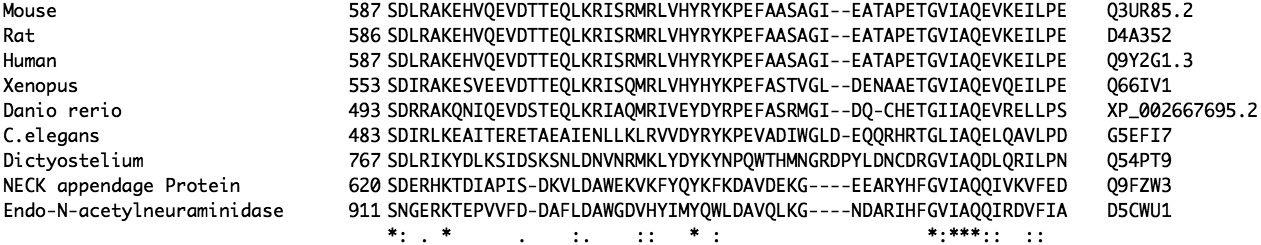

Supplement: Figure S5 — Conservation of the ICD region. Alignment of the ICD for human MYRF/C11Orf9 protein and its orthologs, as well as bacteriophage proteins GA-1 neck appendage protein and Endo-N-acetylneuraminidase, showing conservation of the serine lysine dyad required for cleavage (first and fifth residues in the alignments). (TIF) [file pbio.1001625.s005.tif]
