# Supplementary material for: MYRF Is a Membrane-Associated Transcription Factor That Autoproteolytically Cleaves to Directly Activate Myelin Genes
Source: PLoS Biol. 2013 Aug 13;11(8):e1001625. doi: 10.1371/journal.pbio.1001625 (PMC3742440; doi:10.1371/journal.pbio.1001625)
Supplement: Table S1 — Programs used to predict MYRF protein features. Online programs used to identify the predicted features of the MYRF protein and the associated E-values or scores for each feature are listed. *Values represent E-values unless otherwise stated as being a p value or a score. †Using MYRF residues 546–763 as input. (DOCX) [file pbio.1001625.s007.docx]

| **Feature** | **Search tool** | **Residues** | **E-value or score*** |
| --- | --- | --- | --- |
| Proline-Rich Region | Prosite | 60-330 | Score = 28.785 |
| Ntd80-like DNA Binding Domain | PFAM | 393-540 | 9.00E-28 |
|  | NCBI Conserved Domain Search | 393-536 | 3.16E-36 |
|  | Prosite | 250-541 | Score = 55.754 |
| Transmembrane Domain | TMpred | 768-786 | Score = 2073 (>500 considered significant) |
|  | Das-Tmfilter | 767-787 | 4.480e-04 |
|  | hmmtop | 767-789 | Not given |
|  | Toppred | 767-789 | Score = 2.242  (“certain”) |
| Coiled Coil Domain | Paircoil2 | 672-701 | P=0.0216 |
|  | Prosite | 685-705 | Not given |
| Nuclear Localization Signal | ELM | 253-258 | P=0.0002 |
|  | NucPred | 252-258 | Score=0.88 |
| Intramolecular Chaperone Domain/  Peptidase_S74 Domain | HHpred | 587-689 | 2.80E-17 |
|  | NCBI Conserved Domain Search^†^ | 587-647 | 4.91E-12 |
|  | PFAM | 587-647 | 1.50E-12 |

*Values represent E-values unless otherwise stated as being a P-value or a score

^†^Identified using residues 546-763 as input
