# Supplementary material for: MYRF Is a Membrane-Associated Transcription Factor That Autoproteolytically Cleaves to Directly Activate Myelin Genes
Source: PLoS Biol. 2013 Aug 13;11(8):e1001625. doi: 10.1371/journal.pbio.1001625 (PMC3742440; doi:10.1371/journal.pbio.1001625)
Supplement: Table S4 — Genomic coordinates of genomic regions used for luciferase assays. Genomic coordinates of the regions of the genome (typically ∼700 bp) corresponding to MYRF peaks that were cloned into pGL3-Promoter vector for luciferase assays. The left columns show the genes that these MYRF peaks are associated with and the genes' expression levels in CNS cell types ([10], using the Affymetrix All-Exon dataset). The right column shows the ChIP-Seq signal within each region cloned into pGL3-Promoter (note that the peaks are not necessarily centered in the amplified region as availability of acceptable primer sites was a consideration in determining regions to be cloned). (DOCX) [file pbio.1001625.s011.docx]

**Gene Expression levels** .

| **Gene** | **Neuron** | **Astro** | **OPC** | **Oligo** | **Position of peak relative to TSS** | **Coordinates of amplified region cloned into pGL3-Promoter (UCSC rat 2004)** | **ChIP**  **signal in amplified region** |
| --- | --- | --- | --- | --- | --- | --- | --- |
| *Cntn2* | 503 | 35 | 117 | 3,395 | +4,757 bp  (intronic) | chr13:45,423,710-45,424,296 | 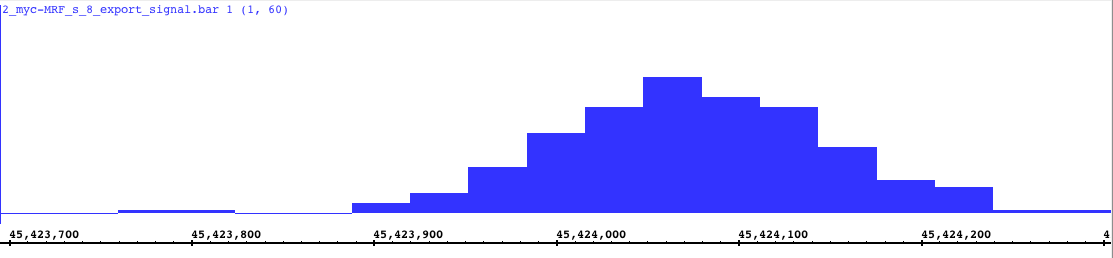 |
| *Cntn2*  *(-ve control region)* | 503 | 35 | 117 | 3,395 | +5,605bp  (intronic) | chr13:45,422,625-45,423,448 | 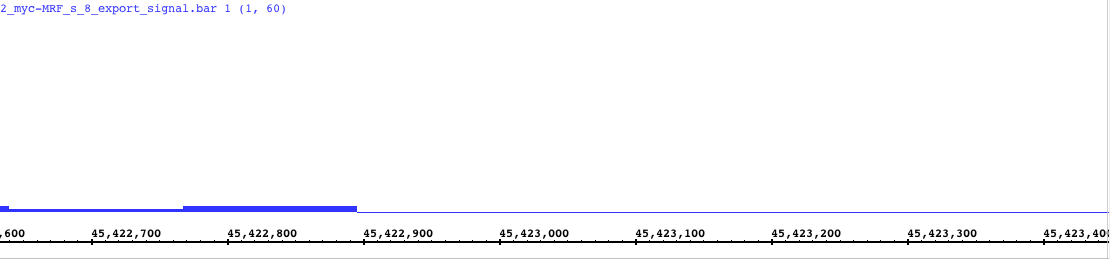 |
| *Tfn* | 107 | 78 | 410 | 14,482 | -313 bp | chr8:108,244,534-108,245,244 | 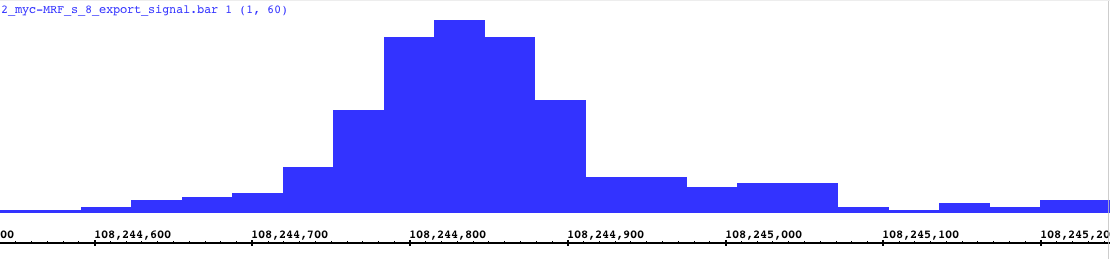 |
| *Mag* | 47 | 46 | 23 | 11,866 | -16 bp | chr1:85,970,642-85,971,235 | 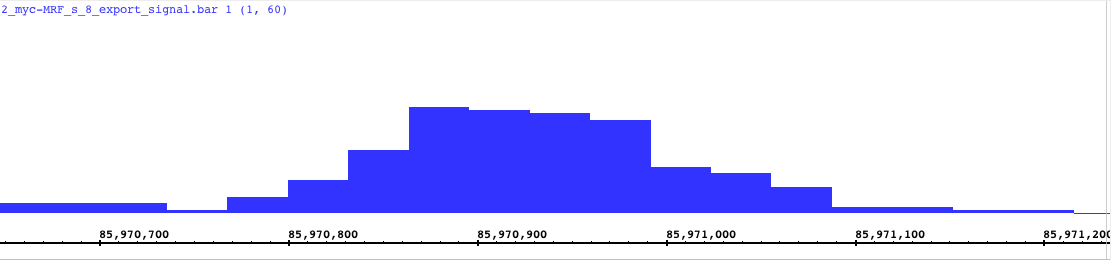 |
| *Mbp* | 478 | 177 | 144 | 10,649 | -19,061 bp | chr18:79,006,011-79,006,944 | 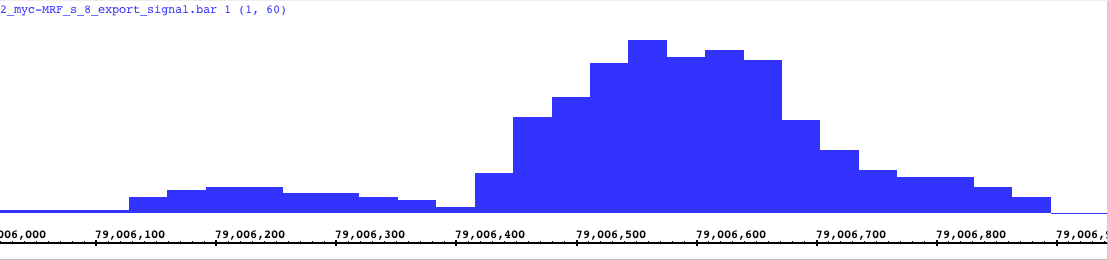 |
| *Plp1* (1) | 907 | 1,680 | 1,242 | 20,798 | -88,697 bp | chrX:124,399,436-124,400,121 | 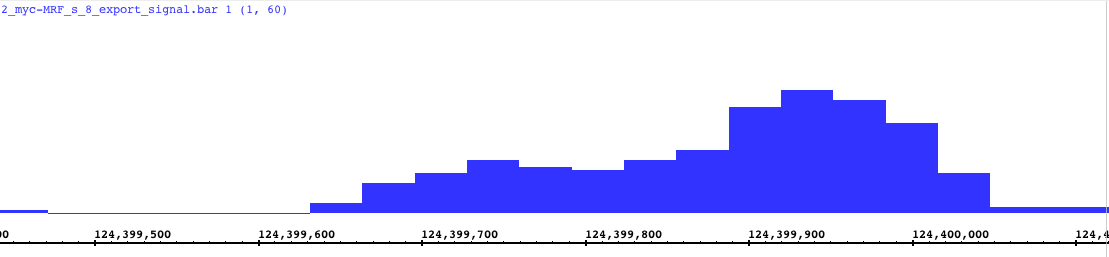 |
| *Plp1* (2) | 907 | 1,680 | 1,242 | 20,798 | -80,697 bp | chrX:124,407,459-124,408,306 | 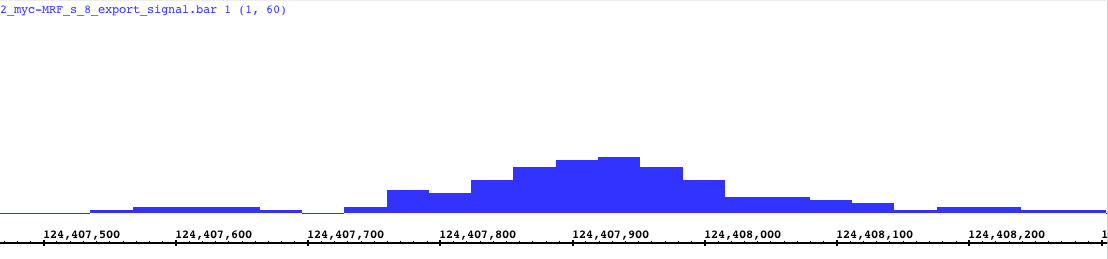 |
| *Rffl* | 41 | 74 | 78 | 2,490 | +12,752 bp  (intronic) | chr10:71,034,165-71,034,749 | 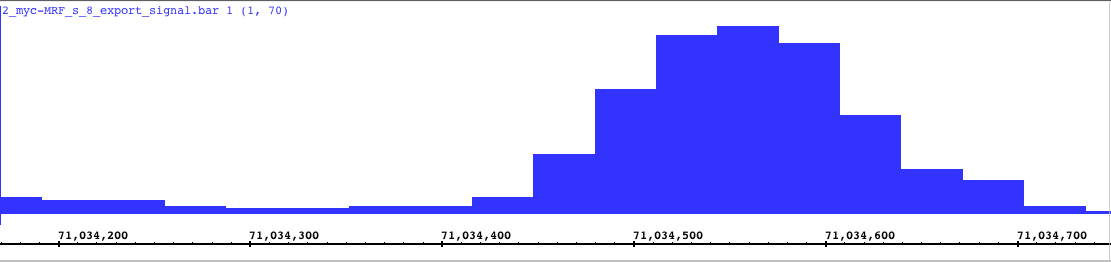 |
| *Nfasc* (1) | 986 | 403 | 1,313 | 11,065 | +10,117 bp  (intronic) | chr13:45,627,390-45,628,388 | 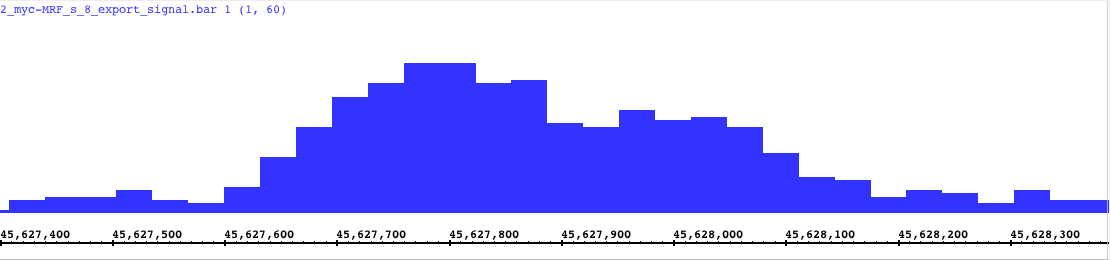 |
| *Nfasc* (2) | 986 | 403 | 1,313 | 11,065 | +34,581 bp  (intronic) | chr13:45,602,970-45,603,778 | 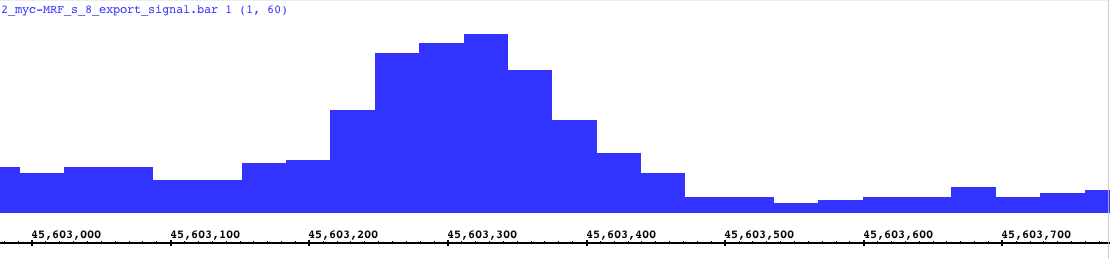 |
