# Supplementary material for: MYRF Is a Membrane-Associated Transcription Factor That Autoproteolytically Cleaves to Directly Activate Myelin Genes
Source: PLoS Biol. 2013 Aug 13;11(8):e1001625. doi: 10.1371/journal.pbio.1001625 (PMC3742440; doi:10.1371/journal.pbio.1001625)
Supplement: Table S5 — Sequences of mutagenesis primers. (DOCX) [file pbio.1001625.s010.docx]

| **Construct** | **Forward Primer (5’-3’)** | **Reverse Primer (5’-3’)** |
| --- | --- | --- |
| MYRF^S587C^ | CTCTTATGCACCCTTGCGATCTGCGGGCCAAG | CTTGGCCCGCAGATCGCAAGGGTGCATAAGAG |
| MYRF^S587A^ | GTTCTCTTATGCACCCTGCCGATCTGCGGGCCAAG | CTTGGCCCGCAGATCGGCAGGGTGCATAAGAGAAC |
| MYRF^K593M^ | GATCTGCGGGCCATGGAGCACGTGCAG | CTGCACGTGCTCCATGGCCCGCAGATC |
| MYRF^K593R^ | GATCTGCGGGCCAGAGAGCACGTGCAG | CTGCACGTGCTCTCTGGCCCGCAGATC |
| MYRF^K593H^ | CGATCTGCGGGCCCATGAGCACGTGCAGG | CCTGCACGTGCTCATGGGCCCGCAGATCG |
| MYRF^K339A^ | GTGTGCCAGAAGGCGAACCACTTCCAGGTG | CACCTGGAAGTGGTTCGCCTTCTGGCACAC |
| MYRF^R453A^ | CACAGTCAGACGCAAGCAAGAGGCCCTTC | GAAGGGCCTCTTGCTTGCGTCTGACTGTG |
| MYRF^R477A^ | CAAAAGTGACCGTGGGGGCGCTCCATTTCAGTGAGAC | GTCTCACTGAAATGGAGCGCCCCCACGGTCACTTTTG |
| MYRF^∆NLS^ | cctccacacccctctgcggcggcgaagcactctgaatca | tgattcagagtgcttcgccgccgcagaggggtgtggagg |
| ∆Cntn2 | CCTGTGCCTGCACCCCTTCACTGAGCAGCTGGCCTGGGCAA | TTGCCCAGGCCAGCTGCTCAGTGAAGGGGTGCAGGCACAGG |
| ∆Mag | CTCCTCTGCCCCCTGGTCGCTGAGGGCCGCCTGCCTCC | GGAGGCAGGCGGCCCTCAGCGACCAGGGGGCAGAGGAG |
| ∆Trf | GTGGCTTGCTCCCGGTCGCTGAGGCTCTGCTTGCCAAC | GTTGGCAAGCAGAGCCTCAGCGACCGGGAGCAAGCCAC |
| ∆Rffl | GTGTGACTACCCCACAAGTCAACATTGCCTGGCGCGGCCACTC | GAGTGGCCGCGCCAGGCAATGTTGACTTGTGGGGTAGTCACAC |
| ∆Nfasc (1) | CAGGCATGGAGCTGGTCAATATTGACTACGCTCGTGAAGGAGGG | CCCTCCTTCACGAGCGTAGTCAATAATGACCAGCTCCATGCCTG |
| ∆Nfasc (2) | GAGGGGATAGATGGAGAGAGATATTGACCTGGACCAGGCTC | GAGCCTGGTCCAGGTCAATATCTCTCTCCATCTATCCCCTC |
